# Eastern Canadian Gastrointestinal Cancer Consensus Conference 2025

**DOI:** 10.3390/curroncol33040228

**Published:** 2026-04-17

**Authors:** Arwa Ahmed, Stéphanie L. Mercier, Ravi Ramjeesingh, Robert Thompson, Donald James Bastin, Silvana Spadafora, Thais Baccili Cury Megid, Vladimir Djedovic, Amandeep S. Taggar, Conrad Falkson, Abdul Rehman Farooq, Gordon Emil Locke, Stacie Connors, Hao Yu Wang, Mustapha Tehfe, Francine Aubin, Setareh Samimi, James Michael, Holly Campbell, Eve St-Hilaire, Suneil Khanna, Mohammed Saud Ali Al Darai, Pierre Whitlock, Angela Hyde, Luisa Galvis, Marie-Philippe Saltiel, Adrian Bailey, Doha Itani, Rakesh Goel, Wadima Aldarmaki, Shivani Dadwal, Rachel Goodwin, Timothy R. Asmis

**Affiliations:** 1 The Ottawa Hospital Cancer Centre, Ottawa, ON K1H 8L6, Canada; arahmed@toh.ca (A.A.); smercier@toh.ca (S.L.M.); dbastin@toh.ca (D.J.B.); vdjedovic@toh.ca (V.D.); golocke@toh.ca (G.E.L.); haowang1@toh.ca (H.Y.W.); maldarai@toh.ca (M.S.A.A.D.); adbailey@toh.ca (A.B.); rgoel@toh.ca (R.G.); waldarmaki@toh.ca (W.A.); rgoodwin@toh.ca (R.G.); 2 Queen Elizabeth II Health Sciences Center, Halifax, NS B3H 3A7, Canada; ravi.ramjeesingh@nshealth.ca; 3 Department of Medicine, Dalhousie University, Saint John, NB E2L 4L5, Canada; robertd.thompson@horizonnb.ca; 4 Algoma District Cancer Program, Sault Ste. Marie, ON P6B 0A8, Canada; spadaforas@sah.on.ca; 5 Dr. Georges-L.-Dumond University Hospital Center, Moncton, NB E1C 2Z3, Canada; thais.megid@vitalitenb.ca; 6 Sunnybrook Health Sciences Centre, Toronto, ON M4N 3M5, Canada; aman.taggar@sunnybrook.ca; 7 Kingston Health Sciences Center, Kingston, ON K7L 2V7, Canada; conrad.falkson@kingstonhsc.ca; 8 Nova Scotia Health Authority, Halifax, NS B3S 0H6, Canada; abdul.farooq@nshealth.ca; 9 Horizon Health Network, Fredericton, NB E3B 4R3, Canada; dr.stacie.connors@horizonnb.ca (S.C.); pierre.whitlock@horizonnb.ca (P.W.); dr.luisa.galvis@horizonnb.ca (L.G.); 10 Centre Hospitalier de l’Universite de Montreal, Montreal, QC H2X 3E4, Canada; mustapha.tehfe.med@ssss.gouv.qc.ca (M.T.); francine.aubin.med@ssss.gouv.qc.ca (F.A.); marie-philippe.saltiel.med@ssss.gouv.qc.ca (M.-P.S.); 11 Hopital du Sacre-Coeur de Montreal, Montreal, QC H4J 1C5, Canada; 12 Saint John Regional Hospital Oncology Center, Saint John, NB E2L 4L2, Canada; james.michael@horizonnb.ca; 13 Horizon’s Saint John Regional Hospital, Saint John, NB E2L 4L2, Canada; holly.campbell@horizonnb.ca (H.C.); doha.itani@horizonnb.ca (D.I.); 14 Vitalité Health Network, Bathurst, NB E2A 1A9, Canada; eve.st-hilaire@vitalitenb.ca; 15 St. Michael’s Hospital, Toronto, ON M5B 1W8, Canada; suneil.khanna@unityhealth.to; 16 Dr. H. Bliss Murphy Cancer Center, St. John’s, NL A1B 3X5, Canada; angela.hyde@nlhealthservices.ca; 17 Department of Oncology, Faculty of Health Sciences, McMaster University, Hamilton, ON L8S 4L8, Canada; dadwal@hhsc.ca

**Keywords:** guidelines, gastroesophageal junction cancer, biomarkers, colorectal cancer, secondary prevention, hepatocellular carcinoma, systemic therapy

## Abstract

The Eastern Canadian Gastrointestinal Cancer Consensus Conference convened annually and was held in Fredericton, New Brunswick, from 18 to 20 September 2025. Attendees included experts in medical oncology, radiation oncology, nuclear medicine, and general practitioners in oncology (GPO) from across the eastern Canadian provinces who are engaged in the care and management of patients with gastrointestinal malignancies. The consensus statement resulting from this meeting addresses several key topics, including the management of early-stage gastroesophageal junction cancer, recent developments in molecular biomarkers and colorectal cancer treatments, secondary prevention strategies for colorectal cancer, and treatment of hepatocellular carcinoma.

## Simple Summary

The Eastern Canadian Gastrointestinal Cancer Consensus Conference was held in Fredericton, New Brunswick, from 18 to 20 September 2025. This annual meeting brought together experts from various oncology disciplines who are specialized in treating gastrointestinal (GI) and hepato-pancreatobiliary (HPB) cancers representing eastern Canadian provinces. The purpose of the conference was to address the evolving landscape in the management and care of gastrointestinal cancers and to develop consensus statements based on the most recent scientific evidence and clinical experience. Ahead of the event, the organizing committee and speakers identified section topics that reflected the most common questions faced by clinicians treating gastrointestinal and hepatobiliary malignancies in eastern Canada. During the conference, presentations highlighted advances in areas such as the multidisciplinary management of early-stage gastroesophageal junction cancer, the integration of molecular biomarkers into clinical decision-making, recent developments in colorectal cancer treatments, secondary prevention strategies for colorectal cancer, and the treatment of hepatocellular carcinoma. Following these presentations, consensus recommendations were developed collaboratively by the participating physicians and categorized by the level of supporting evidence. The consensus statement provides guidance to clinicians in Canada, helping to standardize and improve the quality of care for patients with gastrointestinal malignancies by summarizing expert opinions and evidence-based practices for each topic covered.

## 1. Introduction

The annual Eastern Canadian Gastrointestinal Cancer Consensus Conference was held in Fredericton, New Brunswick, between 18 and 20 September 2025. The objective of this conference was to develop consensus statements on evolving topics in the management of gastrointestinal (GI) cancers. Conference participants included medical oncologists, radiation oncologists, pathologists, and general practitioners in oncology (GPOs) from across the eastern Canadian provinces, including Ontario, Quebec, New Brunswick, Nova Scotia, Newfoundland and Labrador, and Prince Edward Island. Ahead of the conference, the organizing committee and speakers developed section topics representing the most common questions clinicians treating gastrointestinal and hepatobiliary malignancies in eastern Canada face. Each of the following consensus statements were developed following a presentation on the most recent advances from relevant scientific evidence.

The recommendations presented in this paper summarize the consensus opinions for each section by the physicians involved in managing GI and HPB malignancies who participated in the meeting. These recommendations were formulated as a group and categorized by the level of evidence:

• Level I: Evidence from randomized controlled trials.
• Level II-1: Evidence from controlled trials without randomization.
• Level II-2: Evidence from analytic cohorts or case–control studies.
• Level II-3: Evidence from comparisons between times or places with and without the intervention.
• Level III: The opinion of respected authorities based on clinical experience, descriptive.

## 2. Multidisciplinary Management of Early-Stage Gastroesophageal Cancer

Question 1: What are the minimum biomarker requirements for patients with gastroesophageal cancer?

• For early-stage gastroesophageal cancer, we recommend sending for biomarker testing at the time of initial biopsy [Level III].
• All esophageal, gastroesophageal junction (GEJ), and proximal gastric adenocarcinomas must have the following biomarkers tested reflexively and synchronously [Level I]:
  ○ MMR (Mismatch repair).
  ○ HER2 (Human epidermal growth factor 2).
  ○ PD-L1 (Programmed death-ligand 1).
  ○ Claudin 18.2.
• In the advanced or metastatic (non-curative) setting, we support biomarker testing for NTRK (Neurotrophic Tyrosine Receptor Kinase) for esophageal, GEJ, and proximal gastric adenocarcinoma [Level II].
• For esophageal, GEJ, or proximal gastric squamous cell carcinomas, we recommend that, at a minimum, biomarker testing for PD-L1 be performed using CPS (combined positive score) or TAP (tumor area positivity) scoring [Level I].

Question 2: What are the general management principles for patients presenting with a diagnosis of resectable locally advanced gastroesophageal junction cancer?

• We recommend that the management of patients with resectable locally advanced gastroesophageal junction cancer be discussed by a multidisciplinary tumor board (MDT), which should include thoracic/esophageal surgical oncologists, radiation oncologists, medical oncologists, radiologists, pathologists, and gastroenterologists [Level III].
• Medically fit patients with locally advanced gastroesophageal adenocarcinoma should be considered for a perioperative FLOT chemotherapy regimen [Level I].
  ○ The CROSS chemoradiotherapy (CRT) regimen remains an acceptable alternative neoadjuvant approach [Level I].
• The neoadjuvant CROSS CRT regimen remains the standard of care for gastroesophageal squamous cell carcinoma suitable for surgical resection [Level I].

Question 3: What is the optimal perioperative treatment for curative-intent esophageal squamous cell carcinomas?

• We endorse the neoadjuvant CROSS CRT protocol as the standard of care for gastroesophageal squamous cell carcinoma [Level I].
  ○ We endorse using the radiation dose and schedule as per the CROSS trial protocol (41.4 Gy in 23 fractions), with weekly carboplatin/paclitaxel [Level I].
• For patients treated with chemoradiation followed by surgical resection without a pathologic complete response on the surgical specimen, adjuvant Nivolumab may be considered (as guided by the tumor’s PD-L1 CPS) [Level I].

Question 4: What is the optimal perioperative treatment for patients with resectable Gastroesophageal adenocarcinoma?

• Medically fit patients with locally advanced gastroesophageal adenocarcinoma should be considered for a perioperative FLOT chemotherapy regimen [Level I].
  ○ The CROSS chemoradiotherapy (CRT) regimen remains an acceptable alternative neoadjuvant approach for patients [Level I].
• The management of patients with inadequate pathologic response or poor pathologic indicators following neoadjuvant FLOT should be discussed in MDT team [Level III].
• For patients without a contraindication to immunotherapy, a new standard of care may include Durvalumab plus FLOT (D-FLOT) [Level I].
• For patients with esophageal or GEJ adenocarcinoma who are not candidates for FLOT, the CROSS regimen followed by adjuvant Nivolumab (if there is no pathologic complete response and based on PD-L1 CPS) is an option [Level I].

### 2.1. Summary of Evidence

#### 2.1.1. Biomarkers in Gastroesophageal Cancers

The incidence of gastroesophageal junctional (GEJ) cancers and esophageal cancers has been increasing in Western countries over the past few decades, particularly when compared to the incidence of gastric cancers, with 5-year overall survival of less than 30% [1]. The treatment of early-stage or locally advanced GEJ cancers requires a multimodality approach, while the treatment of metastatic disease is now primarily dictated by molecular biomarkers.

Human epidermal growth factor 2 (HER2) testing was the first molecular test introduced in the treatment of metastatic gastric and GEJ cancers. The TOGA clinical trial demonstrated an improved median overall survival (mOS) in patients with HER2 overexpression or amplification with the addition of the anti-HER2 antibody Trastuzumab to chemotherapy 13.8 months versus 11.1 months (HR 0.74; 95% CI 0.60–0.91, *p* = 0.0046) [2]. Testing for HER2 status is performed using immunohistochemistry (IHC) and/or fluorescence in situ hybridization (FISH). In clinical trials, HER2 positivity was defined as an IHC score of 3+ or an IHC score of 2+ with positive FISH, and FISH positivity was defined as a ratio of ≥2.0 for the number of HER2 copies to the number of signals for CEP17.

Programmed death-ligand (PD-L1) is a well-established biomarker in determining the benefit of immune checkpoint inhibitors (ICIs) in combination with chemotherapy in esophageal, GEJ or gastric cancers. Nivolumab and pembrolizumab are both anti-PD-1 monoclonal antibodies that have shown survival benefits (both alone and in combination with chemotherapy) in PD-L1-positive metastatic malignancies, and are now part of the standard of care in the first-line metastatic setting [3,4,5]. The role of immune checkpoint inhibitors in the perioperative management of resectable GEJ and gastric cancers is unfolding with the recent positive results from the MATTERHORN clinical trial (discussed further below). The level of PD-L1 positivity in the tumor cells is measured by different scoring systems. Most published clinical trials on the treatment of gastric, GEJ or esophageal cancers used combined positive score (CPS) to measure the PD-L1, while the tumor area positivity (TAP) score was used in the perioperative MATTERHORN trial [6].

Claudin 18.2 is another targetable molecular biomarker in GEJ cancers, with recent studies demonstrating survival benefits with the addition of anti-Claudin 18.2 antibody therapies to chemotherapy in the metastatic setting [7,8]. Claudin 18.2 testing is performed via immunohistochemistry (IHC), with positivity defined as moderate-to-strong membranous staining in at least 75% of tumor cells.

Mismatch repair deficiency (dMMR), or microsatellite instability-high (MSI-H), correlates with higher response to ICI across different solid tumors. In metastatic or unresectable esophageal, GEJ or gastric cancers, the presence of dMMR/MSI-H is an indication for treatment with combination ICI and chemotherapy independent of PD-L1 expression [9]. In the phase 3 Keynote-062 trial, an exploratory analysis of patients with MSI-H tumors showed that the 12- and 24-month overall survival rates were higher for both the pembrolizumab monotherapy group (79% and 71%, respectively) and the pembrolizumab plus chemotherapy group (71% and 65%, respectively), compared to the chemotherapy alone group (47% and 26%, respectively). In addition, the median progression-free survival numerically favored the combination of pembrolizumab and chemotherapy [10]. In light of these data, pembrolizumab monotherapy may be considered as a treatment option for select patients with advanced dMMR/MSI-H gastric or gastroesophageal junction cancers who are unable to tolerate combination therapy.

In the neoadjuvant space, emerging data from a phase 2 clinical trial demonstrates that dual immune checkpoint inhibition has promising efficacy in dMMR/MSI-H gastric and GEJ cancers, with notable rates of pathological complete response [11]. At present, this remains experimental, and clinical trial participation should be encouraged where available.

We recommend upfront testing for molecular biomarkers in metastatic and early-stage GEJ adenocarcinoma given their aggressive nature and high risk of recurrence. For squamous cell histology, PD-L1 testing is recommended as a minimum requirement before finalizing treatment decisions.

#### 2.1.2. Initial Management and Staging Considerations in Resectable Locally Advanced Gastroesophageal Junction Cancer

Early-stage gastroesophageal junction (GEJ) and gastric cancers are defined as tumors confined to the lamina propria, mucosa or submucosa of the stomach or esophagus (T1 tumors) with no lymph node involvement or distant metastasis. Such cases are frequently managed with endoscopic procedures, including Endoscopic Submucosal Dissection (ESD) and Endoscopic Mucosal Resection (EMR), or through surgical intervention. Tumors with more locally advanced tumor stage (T2 or higher) or associated with lymph nodes involvement in the absence of distant spread typically warrant perioperative therapy.

A multidisciplinary approach to the management of all patients with potentially curable disease is integral to optimizing patient outcomes and survival. Given the aggressive nature of gastroesophageal cancers, with 50% of becoming non-curative during their clinical course, we recommend testing for relevant biomarkers (outlined above under Question 2) at the time of initial biopsy confirmation of gastroesophageal cancer.

The initial staging investigations of patients with localized or locally advanced gastroesophageal cancer should include imaging with a CT scan of thorax and of the abdomen and pelvis, and ideally a PET scan in esophageal and GEJ cancers (though timely access to PET imaging may be challenging in some jurisdictions). Endoscopic ultrasound (EUS) and surgical exploration with staging laparoscopy may be performed as part of the staging work-up for gastroesophageal junction (GEJ) and esophageal cancers, in accordance with discussions held with the surgical team within the institution. Restaging imaging upon completion of preoperative/neoadjuvant systemic therapy (with CT or PET/CT) should be considered prior to surgery. Patients who present with locoregional recurrence following primary treatment should undergo restaging imaging, preferably with PET/CT if available, when definitive salvage therapy is being considered.

#### 2.1.3. Perioperative Chemotherapy and Chemoradiation in Gastroesophageal Cancer

In 2006, the phase III randomized MAGIC trial demonstrated that perioperative ECF (epirubicin, cisplatin, and fluorouracil) improved overall survival at 5 years when compared to surgery alone (36% versus 23%; HR 0.75, 95% CI 0.60–0.93, *p* = 0.009) in patients with adenocarcinoma of the stomach, gastroesophageal junction, or lower esophagus [12]. In 2011, a smaller French trial evaluated perioperative intravenous fluorouracil + cisplatin versus surgery alone in a similar patient population; in this trial, perioperative systemic therapy also improved 5 year overall survival (38% versus 24%; HR 0.69, 95% CI 0.50–0.95, *p* = 0.02) [13].

In 2012, van Hagen et al. published the CROSS chemoradiation protocol [14]. Patients with both adenocarcinoma (75% of the overall cohort) and squamous cell carcinoma (23% of the overall cohort) of the esophagus or gastroesophageal junction (stage T1 N1 or T2–3 N0–1) were included and randomized to chemoradiotherapy followed by surgery versus surgery alone. Chemoradiotherapy consisted of intravenous systemic chemotherapy (Carboplatin at an AUC of 2 mg/mL/min and Paclitaxel at 50 mg/m^2^) administered on days 1, 8, 15, 22 and 29, as well as a total radiation dose of 41.4 Gy administered in 23 fractions of 1.8 Gy each; 5 fractions were administered per week, beginning on the first day of the first treatment of chemotherapy. In the intention-to-treat cohort, the median overall survival (mOS) was 49.4 months in the chemoradiation group versus 24.0 months in the surgery group (HR 0.657; 95% CI 0.495–0.871, *p* = 0.003). While both groups did benefit, when analyzed by histologic subgroup, the magnitude of benefit was greater among patients with squamous cell carcinoma (mOS 81.6 months versus 21.1 months [HR 0.48; 95% CI 0.28–0.83]) than in those with adenocarcinoma (mOS 43.2 months versus 27.1 months [HR 0.73; 95% CI 0.55–0.98]) in favor of neoadjuvant chemoradiation.

Subsequently, the German phase 2/3 FLOT4 trial randomized patients with locally advanced and resectable gastric or gastroesophageal adenocarcinoma to FLOT (docetaxel, oxaliplatin, leucovorin, and intravenous fluorouracil infused over 24 h) administered every two weeks for four cycles preoperatively and for four cycles postoperatively, or to ECF/ECX (as per the MAGIC chemotherapy protocol) administered every three weeks for three cycles preoperatively and for three cycles postoperatively. The median overall survival was significantly increased in the FLOT group (50 months compared to 35 months in the ECF/ECX group [HR 0.77; 95% CI 0.63–0.94, *p* = 0.012]). The 5-year overall survival rate was 45% in the FLOT group compared to 36% in the ECF/ECX group [15]. These results established perioperative FLOT chemotherapy as the standard of care for nonmetastatic potentially resectable gastric cancer and for gastroesophageal junctional cancers (which represented 56% of enrolled patients in the FLOT trial, compared to 24% in the CROSS trial).

#### 2.1.4. Perioperative Systemic Therapy Versus Preoperative Chemoradiation

Three major trials have since sought to compare perioperative systemic therapy to neoadjuvant chemoradiation for the management of localized esophageal, gastric and/or gastroesophageal adenocarcinoma—Neo-AEGIS, ESOPEC, and TOPGEAR.

The European Neo-AEGIS trial, which was still recruiting as the FLOT4 trial data was becoming available, compared trimodality therapy (pre-operative chemoradiation, as defined in the CROSS regimen) to perioperative chemotherapy—consisting either of a modified MAGIC regimen (ECF/EOX) in those recruited prior to 2018—or to FLOT in those recruited after 2018. Neo-AEGIS enrolled patients with esophageal and gastroesophageal junction (Siewert types I, II and III). Approximately 14% of patients in the perioperative chemotherapy arm received FLOT, with the remainder receiving ECF/EOX. Neo-AEGIS was ultimately underpowered and closed early, following the second futility analysis and due to the impact of the COVID-19 pandemic. Within their dataset, a similar 3-year overall survival and no significant differences in health-related quality of life or operative outcomes were seen [16].

Shortly after this, the results of the German ESOPEC trial were published. ESOPEC randomized patients with locally advanced esophageal and gastroesophageal junction adenocarcinoma to perioperative FLOT or to preoperative chemoradiotherapy (CROSS). The median overall survival in the FLOT group was 66 months (95% CI 36 months—not reached), compared to 37 months in the chemoradiotherapy group (95% CI 28 months—43 months), establishing the superiority of perioperative chemotherapy over preoperative chemoradiation for patients with cT1 cN+ or cT2-4a cN0/cN+ gastroesophageal adenocarcinoma (HR 0.70; 95% CI 0.53–0.92, *p* = 0.01) [17].

The phase III TOPGEAR trial sought to determine if the addition a course of chemoradiation to perioperative chemotherapy might improve outcomes. Patients with gastric or gastroesophageal junction adenocarcinoma were randomized to either ECF or FLOT alone, or to ECF or FLOT with the addition of chemoradiation (the radiation dose was specified at 45 Gy in 25 fractions delivered with infusional 5-fluorouracil). After a median follow up of 67 months, no significant between-group differences in progression-free survival and in overall survival were noted [18].

To summarize the above data, the benefit of perioperative chemotherapy in early-stage gastroesophageal cancer is now well established. For patients with locally advanced resectable esophageal adenocarcinoma, the contemporaneous ESOPEC trial has confirmed that perioperative FLOT is superior to preoperative chemoradiotherapy in those who are fit enough to undertake this regimen; otherwise, preoperative chemoradiation regimen as per the CROSS protocol remains a reasonable alternative. The CROSS protocol holds the highest level of evidence for management of locally advanced and resectable esophageal squamous cell carcinoma.

#### 2.1.5. Beyond Chemoradiation: Immunotherapy and Future Directions

Achieving a pathologic complete response following the receipt of preoperative therapy is an established prognostic factor in adenocarcinomas of the esophagus and the gastroesophageal junction. In the CHECKMATE-577 trial, patients with adenocarcinoma or squamous cell carcinoma of the esophagus or gastroesophageal junction having received neoadjuvant chemoradiation and who were found to have residual pathologic disease following R0 surgical resection (≥ypT1 or ≥ypN1) were randomized to receive nivolumab, a PD1-blocking monoclonal antibody, or placebo for up to one year. At 24 months, the median disease-free survival in the nivolumab group was of 22.4 months [95% CI 16.6–24.0] compared to 11.0 months [95% CI 8.3–14.3] in the placebo arm (HR for disease recurrence or death, 0.69) [19]. A first overall survival update was presented at the recent American Society of Clinical Oncology (ASCO) meeting in June 2025; after 78.3 months of follow-up, there was no statistically significant improvement in overall survival. The updated mOS was 51.7 months (95% CI 41.0–61.6) in the nivolumab group versus 35.3 months (95% CI 30.7–48.8) in the placebo arm (HR 0.85; 95% CI 0.70–1.04, *p* = 0.1064) [20]. When stratified by the tumor’s PD-L1 CPS, for tumors with a PD-L1 CPS ≥ 1, the mOS was 45.5 months with nivolumab versus 33.5 months in the placebo group (HR 0.79; 95% CI 0.64–0.99); meanwhile, for tumors with a PD-L1 CPS < 1, the mOS in the nivolumab arm was of 39.2 months versus 52.8 months in the placebo arm (HR 1.40; 95% CI 0.77–2.56) [20].

Two recent trials have looked at intensifying perioperative chemotherapy with the addition of immunotherapy: KEYNOTE-585 and MATTERTHORN.

The KEYNOTE-585 trial randomized patients with resectable T3/T4 or node-positive GEJ or gastric adenocarcinoma to perioperative chemotherapy (intravenous cisplatin in combination with either oral capecitabine or intravenous fluorouracil in the main cohort of 804 patients; an additional 203 patients on FLOT were subsequently included) with either pembrolizumab 200 mg IV every 3 weeks or placebo; on completion of the perioperative chemotherapy, pembrolizumab or placebo were continued for 11 cycles [21]. At the time of final analysis, no overall survival advantage was observed over Ppacebo with the addition of pembrolizumab in either the main cohort or in the smaller FLOT cohort [22].

The MATTERHORN trial randomized patients with resectable stage II to IVa gastric or GEJ adenocarcinoma to receive either 2 cycles of neoadjuvant durvalumab (1500 mg IV every 4 weeks) or 2 cycles of placebo every 4 weeks, in addition to four cycles of FLOT (every 2 weeks) followed by two doses of adjuvant durvalumab or placebo (every four weeks) with four cycles of FLOT (every 2 weeks). Durvalumab or placebo were then continued to complete up to one year of adjuvant therapy. The addition of durvalumab to FLOT resulted in a statistically significant improvement in event free survival when compared to placebo with FLOT [HR 0.71; 95% CI 0.58–0.86; *p* < 0.001]; the median EFS has not yet been reached in the Durvalumab + FLOT cohort (95% CI 40.7 mo–NR], compared to 32.8 months in the Placebo + FLOT group [95% CI 27.9 mo–NR] [23]. This benefit has been preserved across subgroups, including in the TAP < 1% group [HR 0.77; 95% CI 0.40–1.46], acknowledging the wide 95% confidence interval. The final overall survival analysis of MATTERHORN was recently presented at the 2025 ESMO Congress in Berlin, Germany. The results revealed that the combination of Durvalumab + FLOT reduced the risk of death by 22% (HR 0.78; 95% CI 0.63–0.96, *p* = 0.021) when compared to FLOT alone. The median overall survival has not been reached in either arm [6].

Of note, the FLOT comparator arms in the KEYNOTE-585 and MATTERHORN trials underperformed relative to the intention-to-treat cohort in the original FLOT4 trial. This may be due to inclusion of larger tumors in both KEYNOTE-585 (where cT3/cT4 tumors made up 95% of the Pembrolizumab + FLOT cohort and 93% of the FLOT comparator arm) and MATTERHORN (where cT3/T4 tumors made up 89% of the ITT cohort versus 92% of the FLOT cohort); for contrast, in FLOT4, cT3/T4 tumors comprised 82% of the ECF cohort and 81% of the FLOT cohort. These larger tumors are more difficult to downstage with neoadjuvant systemic therapy and may pose greater challenges for surgical resection.

Based on these data, the addition of adjuvant nivolumab may be considered for patients who received neoadjuvant chemoradiation for a diagnosis of gastroesophageal cancer whose tumors did not achieve a pathologic complete response, particularly in tumours with a squamous histology and a CPS ≥1, taking note of improved disease-free survival but no significant overall survival improvement in the overall study cohort. It remains uncertain if those with higher CPS (i.e., CPS > 5, CPS > 10) may achieve a greater margin of benefit with adjuvant nivolumab. For patients with gastroesophageal adenocarcinoma who are candidates for perioperative FLOT, the addition of durvalumab may soon become a new standard of care.

## 3. Colorectal Cancer: Biomarkers and Treatment

Question 1: What are the Molecular Biomarkers required for the management decision in Colorectal Cancer (CRC)?

• The minimum standard biomarkers for prognostication and treatment decisions for CRC include the following [Level I]:
  ○ MMR (Mismatch repair).
  ○ KRAS (Kirsten Rat Sarcoma viral oncogene homolog).
  ○ NRAS (Neuroblastoma Ras viral oncogene homolog).
  ○ BRAF (B-Raf serine/threonine kinase).
  ○ PIK3CA/PTEN/PIK3R1 (phosphatidylinositol-4,5-bisphosphate 3-kinase catalytic subunit alpha/Phosphatase and Tensin homolog/Phosphoinositide-3-Kinase Regulatory Subunit 1).
• For advanced metastatic CRC, the following biomarkers are needed prior to the initiation of med oncology consultation: MMR, RAS and BRAF [Level I].
• PIK3CA/PTEN/PIK3R1 mutation are the biomarkers needed to discuss the use of ASA in stage 1–3 CRC [Level I].
• For advanced/metastatic CRC, we endorse testing for potentially actionable mutations, including NTRK and HER2 [Level II].
• We recommend that these biomarkers be tested reflexively and synchronously [Level III].

Question 2: What biomarker testing should be performed to define the role of Immune Checkpoint Inhibitors (ICIs) in colorectal cancer?

• All patients with a diagnosis of colorectal cancer should have either IHC (Immunohistochemistry) testing for MMR protein or PCR/NGS (polymerase chain reaction/next-generation sequencing) for MSI (microsatellite instability) performed [Level I].

Question 3: Should patients with advanced unresectable, metastatic dMMR/MSI-H (deficient mismatch repair/microsatellite instability-High) colorectal cancer receive ICI in the first-line setting?

• First-line therapy with ICI is preferred over a chemotherapy-based approach in the absence of medical contraindication to ICI [Level I].
• Options for single agent pembrolizumab or dual immunotherapy with nivolumab and ipilimumab are both appropriate [Level I].
• Our preference is dual ICI; however, we acknowledge that this decision needs to be made in the context of disease and patient factors. This discussion should include differences in upfront resistance, expected response rate, adverse events profiles, patient clinical performance status, and comorbidities [Level III].

Question 4: What is the role of immunotherapy in the adjuvant setting for dMMR/MSI-H colon cancer?

• In the adjuvant setting, atezolizumab + oxaliplatin-based chemotherapy is the preferred option for resected stage III dMMR/MSI-H colon cancer [Level I].

Question 5: For pMMR (proficient mismatch repair) metastatic CRC, what is the new practice-changing data for first-line systemic treatment decision-making?

• Doublet chemotherapy with encorafenib and an anti-EGFR (anti-epidermal growth factor receptor) agent (such as Cetuximab or Panitumumab) is recommended in the first-line setting for pMMR BRAF V600E mutation mCRC [Level I].
  ○ We endorse the use of chemotherapy plus encorafenib and anti-EGFR as second-line therapy after immune checkpoint inhibitor (ICI) in the first-line setting for patients with dMMR BRAF V600E mutant mCRC.

### 3.1. Summary of Evidence

#### 3.1.1. An Overview of Key Molecular Biomarkers in Colorectal Cancer

Many molecular alterations have been identified to play a significant role in the pathogenesis and proliferation of CRC. Among these, only few biomarkers have known prognostic and/or predictive values and can therefore be used to inform treatment decisions. We recommend testing for MMR/MSI, KRAS, NRAS, BRAF, PIK3CA and PTEN at minimum before making treatment decisions in CRC.

Mismatch repair deficiency (dMMR) or high level of microsatellite instability (MSI-H) is found in approximately 15% of CRC. It is most commonly detected in early stage disease, and is only identified in 3.8% of metastatic CRC cases [24]. Testing for dMMR/MSI CRC is essential and should be performed upon diagnosis at any stage of the disease using IHC, PCR or NGS. Germline mutation in MMR genes is pathognomonic for Lynch syndrome, making it important for risk stratification and screening for the patients and their relatives. Early-stage dMMR/MSI-H CRC is known to have better prognosis and outcomes compared to their matched-stage pMMR/MSS CRC, while in advanced stage CRC, MSI-H tumors may have poor outcomes compared to MSS CRC [25]. Additionally, dMMR/MSI-H is a strong predictive marker for response to ICI in CRC.

Mutations in the rat sarcoma virus (RAS) family of oncogenes are key drivers for proliferation and survival of CRC cells. Kirsten RAS (*KRAS*) is the most frequently mutated oncogene in CRC and is found in approximately 45% of cases, while Neuroblastoma RAS (NRAS) is found in only around 4–5% of cases [26]. These mutations are most frequently detected in codon 12, 13, 59, 61, 117 and 146, with codon 12 representing the majority of KRAS mutations and codon 61 representing the majority of NRAS. Collective data has shown that most KRAS and NRAS mutations negatively impact the prognosis of advanced CRC and are linked to a more aggressive disease course. In a randomized phase 3 trial, the median OS of advanced CRC was significantly lower in extended RAS mutant CRC compared to wild-type CRC (25 months versus 32.1 months) [27]. RAS mutations prevent hydrolyzation of guanosine triphosphate (GTP) to guanosine diphosphate (GDP), resulting in persistent activating of the MAPK and PI3K/AKT pathway independent of epidermal growth factor (EGFR) receptor signaling, making the presence of one of these mutations a strong predictive marker for lack of response to anti-EGFR therapies. In a retrospective analysis of the CRYSTAL trial, Cetuximab combined with FOLFIRI chemotherapy showed improvement in mOS in KRAS wild-type advanced CRC 24.9 months compared to 21 months in the KRAS mutant group (HR 0.84; 95% CI 0.64–1.11) [28]. With expanded testing for RAS mutations, additional improvement in mOS with Cetuximab was reported in all RAS wild-type cancers (28.4 months versus 20.2 months in RAS mutant malignancies [HR 0.69; 95% CI 0.54–0.88]) [29]. Similar results were seen in the PRIME trial, which looked at Panitumumab combined with FOLFOX chemotherapy in KRAS wild-type advanced CRC compared to FOLFOX alone. Improvements in mPFS (10 months versus 8.6 months [HR 0.80; 95% CI 0.67–0.95]) and mOS (23.9 months versus 19.7 months [HR was 0.88; 95% CI 0.73–1.06]) were seen with the Panitumumab combination [30]. We recommend upfront testing for expanded RAS mutations in advanced or metastatic CRC prior to the initiation of systemic therapy to identify patients who may benefit from anti-EGFR therapy.

Targeting KRAS mutation has become a focus of interest in recent years. Sotorasib and Adagrasib are oral selective inhibitors of KRAS G12C mutation that have shown modest activity as monotherapy in metastatic CRC with mutant KRAS G12C, with ORR of 9.7% (95% CI 3.6–19.9) and 19% (95% CI 8–33) respectively [31,32]. More promising results were reported with these agents when combined with an anti-EGFR agent; the combination Sotorasib and Panitumumab showed an ORR of 26% (95% CI 15.3–40.3), while the combination of Adagrasib and Cetuximab showed an ORR of 46% (95% CI 28–66) [32,33]. Better clinical activity is seen with these agents in lung cancers harboring a KRAS G12C mutation, highlighting the possibility of other mutations, molecular alterations, or oncogenes implicated in the resistance mechanisms and low response rates in KRAS G12C mutant CRC [34,35]. Importantly, mutations in KRAS G12C represent only 3–4% of all KRAS mutations seen in CRC, underscoring the need to explore other potential targets [36].

BRAF is another modulator in the MAPK signaling pathway in CRC, and mutations in BRAF occur in around 10–12% of CRC cases [37]. The majority of BRAF mutations occur in codon 600, with BRAF V600E being the most common mutational subtype, while non-V600E mutations represent less than 22% of mutated BRAF oncogenes [38]. BRAF V600E mutation is a negative prognostic biomarker in CRC, and it is associated with more aggressive disease behavior in the early stages, significantly shortening overall survival (OS) and progression-free survival (PFS) in the advanced stages compared to wild-type or non-V600E BRAF mutant CRC [39,40]. BRAF V600E mutations are an independent predictor of resistance to anti-EGFR therapy in RAS wild-type CRC [41]. Oncogenic mutations in BRAF lead to hyperstimulation of the MAPK pathway, resulting in uninhibited cell proliferation, migration and survival independent of EGFR receptor signaling. BRAF inhibitors failed to demonstrate significant efficacy as monotherapy in BRAF mutant CRC, in contrast to BRAF mutant melanoma. Better results were seen with the combination of BRAF inhibitors and anti-EGFR therapy (with or without MEK inhibitors), establishing combination BRAF and EGFR inhibition as the standard-of-care second-line therapy in BRAF V600E mutant CRC [42]. Recently, the addition of chemotherapy to combined BRAF inhibitor and anti-EGFR therapy is emerging as a new first-line therapy in advanced BRAF V600E mutant CRC. We recommend upfront testing for BRAF mutation in advanced or metastatic CRC, before the initiation of systemic therapy, to identify patients with resistance to anti-EGFR therapy upfront as well as those who may benefit from the combination of a BRAF inhibitor and anti-EGFR therapy with or without chemotherapy.

PIK3CA mutations are present in 15–20% of CRC cases; these are associated with cancer cell proliferation, invasion and angiogenesis [43]. Recently, PIK3CA mutations have gained clinical importance through predicting the benefit of using Aspirin (ASA) in resected CRC; ASA 160 mg orally daily led to a lower incidence of recurrence in CRC harboring mutation in PIK3CA, PIK3R1 or PTEN [44]. We recommend upfront testing for PIK3CA, PIK3R1 and PTEN in resected stage 1–3 CRC to identify patients who may benefit from daily low-dose Aspirin.

There are other molecular alterations involved in carcinogenesis, progression and resistance mechanisms in CRC. The most important ones are those identified as actionable targets with the potential of opening the door for treatment options in later lines, such as HER2 and NTRK. Next-generation sequencing (NGS) provides comprehensive information on the molecular landscape of CRC by rapidly and simultaneously sequencing millions of DNA and RNA fragments, and it can be performed on tumor tissue or blood samples (liquid biopsy). Reflex molecular testing is an important and cost-effective way to provide information in a timely manner to medical oncologists.

Human epidermal growth factor 2 (HER2) overexpression or amplification is present in about 5–6% of CRC [45]. These alterations are mostly seen in left-sided RAS and BRAF wild-type tumors. There is association between HER2 alterations and acquired resistance to anti-EGFR therapy, with greater prevalence of HER2 amplification seen upon failure in anti-EGFR therapy; although the mechanism behind this is not well understood, it is reasonable to test for HER2 after treatment failure [46]. HER2 overexpression or amplification is a predictor for response to anti-HER2-targeted therapy; different combinations of anti-HER2 agents have shown meaningful clinical activity and promising results in chemo-refractory HER2-positive KRAS and wild-type metastatic CRC, with objective response rate (ORR) ranging between 30 and 40% [47,48,49]. Trastuzumab deruxtecan (T-DXd) as monotherapy demonstrated improved outcomes in the DESTINY-CRC01 trial, with an ORR of 45.3% (95% CI 31.6–59.6), a mPFS of 6.9 months (95% CI 4.1–8.7) and a mOS of 15.5 months (95% CI 8.8–20.8) in patients with previously treated HER2-positive metastatic CRC, including patients who had progressed in other anti-HER2 therapies, highlighting that T-DXd can overcome resistance to prior anti-HER2 therapy in CRC [50]. In DESTINY-CRC02, T-DXd showed activity in both KRAS wild-type and mutant HER2-positive metastatic CRC [51]. The ongoing MOUNTAINEER-03 randomized phase 3 trial is looking at the efficacy of dual anti-HER2 therapy (Tucatinib and Trastuzumab) in combination with chemotherapy for metastatic HER2-positive KRAS wild-type CRC in the first-line setting (NCT05253651) [52]. HER2 mutation occurs approximately in 4–5% of CRC, and it is indicative of worse overall survival [53]. It is commonly identified concurrently with MSI, PIK3CA mutation and high tumor mutation burden, and it is associated with resistance to anti-HER2 therapy [54]. Testing for HER2 aberrations can be performed with NGS for HER2 amplification and mutations, or by immunohistochemistry (IHC) for HER2 overexpression and fluorescence in situ hybridization (FISH) for amplification.

Neurotrophic Tropomyosin Receptor Kinase (NTRK) rearrangements are rare in CRC, representing 0.2–0.4% of cases, with NTRK1 and NTRK3 being the most reported in the literature [55]. There is a higher incidence of NTRK rearrangement observed in MSI-H mCRC, with an estimated incidence of 5.3% [56]. NTRK rearrangements have been identified in multiple different solid tumors, each with very good response to TRK inhibitors in clinical trials. Larotrectinib is a selective TRK inhibitor and the first in this class to show impressive results in solid tumors with NTRK rearrangements, with a pooled analysis of three phase 1/2 clinical trials with 153 patients (including 8 with CRC), demonstrating an ORR of 79% (95% CI 72–85), a median duration of response (DOR) of 35.2 months (95% CI 22.8—not estimable) and a median OS of 44.4 months (95% CI 36.5—not estimable) [57]. In a recent analysis of 44 patients with NTRK rearrangement positive gastrointestinal cancers treated with Larotrectinib (including 26 cases of CRC), the results showed an ORR of 44% (95% CI 24–65) with a median DOR of 27 months (95% CI 6—not estimable) and a mOS of 29 months (95% CI 7—not estimable) in the CRC subgroup [58]. Testing for NTRK rearrangements can be performed with IHC as an initial screening test, but needs to be confirmed with FISH or, preferably, NGS.

Treatment of metastatic colorectal cancer (mCRC) becomes challenging following progression on standard chemotherapy regimens. It is advisable to perform next-generation sequencing (NGS) using tumor tissue or liquid biopsy, where feasible, to inform subsequent treatment decisions.

#### 3.1.2. The Role of Immune Checkpoint Inhibitors (ICIs) in Colorectal Cancer

Mutation in mismatch repair (MMR) gene causes microsatellite instability (MSI), resulting in the accumulation of DNA mutations within the cancer cells. This leads to an increase in the neoantigens presented on the tumor cell surface, creating a tumor microenvironment rich with immune cell infiltration, enhancing the tumor’s immune response. The loss of MMR proteins (MLH1, PMS2, MSH2 and MSH6) can be tested for using IHC, which is an affordable and widely used technique. Lack of nuclear staining of one or more of the MMR proteins indicates MMR deficiency (dMMR). Microsatellite instability (MSI) can be detected by polymerase chain reaction (PCR), which can identify changes in the length of DNA fragments at microsatellite loci within tumor cells. NGS is another reliable method for MSI testing. As a practical guide, IHC for MMR is recommended in all CRC as an initial testing method, and it can be confirmed when needed by PCR or NGS.

CRC with dMMR or MSI exhibit unique clinical characteristics, with low response to chemotherapy, impressive and durable response to ICI in advanced stage, and better prognosis (especially in early-stage disease), highlighting the importance of identifying MMR/MSI status in all stages of CRC. Treatment with ICI has failed to show benefits in unselected patients with mCRC. Over the years, many studies have shown that dMMR or MSI is a strong predictor for response to ICI in CRC. Single-agent Nivolumab, a program death 1 (PD1) inhibitor, showed clinical activity in dMMR/MSI advanced previously treated CRC with ORR 30% (95% CI 20·8–42·9) [59]. Better results were seen when combined with Ipilimumab, a cytotoxic T-Lymphocyte Antigen 4 (CTLA4) inhibitor, with an ORR of 65% (95% CI 55–73%) and disease control rate of 81% (95% CI 72–87%) at 50.9 months [60]. Forty-eight percent of enrolled patients had ongoing responses at the time of data cut-off. In the KEYNOTE-146 trial, pembrolizumab, a PD-L1 inhibitor, showed comparable results in previously treated dMMR/MSI advanced CRC, with an ORR of 34.9% (95% CI, 23.3–48.0%) and a mOS of 47 months (95% CI, 19.2–not reached) [61]. Notably, the investigators reported that 30% plateaued in OS and PFS at 5 years of follow-up. Results from these trials first led to the approval of ICIs in the second line for treatment of dMMR/MSI mCRC, followed by first-line treatment of dMMR/MSI mCRC, and now they are treatment options in the adjuvant setting for dMMR/MSI CRC.

#### 3.1.3. Immune Checkpoint Inhibition in Advanced Unresectable or Metastatic dMMR/MSI-H Colorectal Cancer

The remarkable success of ICI in the second-line treatment of dMMR/MSI mCRC led to the introduction of these agents as first-line therapies. KEYNOTE-177 randomized phase 3 clinical trial compared single-agent pembrolizumab, a cell programmed death inhibitor (anti-PD1), to standard of care chemotherapy with or without an anti-VEGF or anti-EGFR agent (bevacizumab or cetuximab) as first-line treatment for dMMR/MSI mCRC. The mPFS for the pembrolizumab group was 16.5 months, versus 8 months in the chemotherapy group (HR 0·59; 95% CI 0·45–0·79), and the median overall survival (mOS) was 77.5 months with pembrolizumab versus 36.7 months with chemotherapy (HR 0.73; 95% CI 0.53–0.99). The ORR was 45.1% (95% CI 37.1–53.3) with pembrolizumab versus 33.1% (95% CI 25.8–41.1) with chemotherapy, while the mDOR was 75.4 months with pembrolizumab versus 10.6 months with chemotherapy [62]. At the final analysis, 76% of patients in the pembrolizumab group had ongoing response at 36 months compared to 24% in the chemotherapy group and the estimated 36 months OS rate was 61.4% (95% CI 53.2–68.6%) in the pembrolizumab group compared to 50.3% (95% CI 42.0–58.0%) in the chemotherapy group [62]. Approximately 13% reported complete responses in the pembrolizumab arm, compared to 3.9% in the chemotherapy arm. Additionally, 37% of the study patients had crossed over to pembrolizumab after progression on chemotherapy as per the trial protocol, and a further 25.3% of patients crossed over off protocol (for an effective crossover rate of 62%), which may in part explain the good results in the chemotherapy arm.

The combination of nivolumab (anti-PD1) and ipilimumab cytotoxic T-lymphocyte antigen-4 inhibitor (anti-CTLA-4) was investigated in the CheckMate 8HW phase 3 randomized clinical trial against chemotherapy with or without a biological agent, and nivolumab as monotherapy in patients with dMMR/MSI unresectable or mCRC. In comparison to chemotherapy in the first-line setting, the mPFS was not reached in the nivolumab and ipilimumab arm (compared to 5.8 months [95% CI 4.4–7.8 months] in the chemotherapy arm) [63]. The PFS rates at 12 months and 24 months were significantly better in the nivolumab and ipilimumab arm at 79% (95% CI 72–84%) and 72% (95% CI 64–79%), respectively, compared to 21% (95% CI 11.2–32.0%) and 14% (95% CI 6–25%, respectively, in the chemotherapy arm. Interestingly, in CheckMate 8HW, the combination of nivolumab and ipilimumab showed superior results in comparison to the nivolumab monotherapy arm across different lines of therapy and in immunotherapy naïve patients. The PFS rates at 12 months, 24 months and 36 months were 76%, 71% and 68%, respectively, in the combination arm compared to 63%, 56% and 51%, respectively, in the monotherapy nivolumab arm. The overall response rate was 71% (95% CI, 65–76%) versus 58% (95% CI, 52–64%) in favor of the combination nivolumab and ipilimumab over nivolumab monotherapy [64]. Analysis is ongoing in this study, and OS and ORR are pending.

Both KEYNOTE-177 and CheckMate 8HW showed that ICIs have good safety profiles, with lower rate of adverse events in comparison to chemotherapy. The grade 3 or higher treatment-related adverse events were 22% with pembrolizumab versus 66% with chemotherapy in KEYNOTE-177, and 23% with nivolumab and ipilimumab versus 48% with chemotherapy in CheckMate 8HW. When comparing nivolumab monotherapy to combination nivolumab and ipilimumab in CheckMate 8HW, the reported grade 3 or higher treatment-related adverse event rate were 14% versus 22%, demonstrating a better safety profile with single-agent nivolumab compared to the combination, but keeping in mind that the analysis included patients previously treated but immunotherapy naïve.

Immune-related adverse events (irAEs) are of special concern with ICI therapy with growing attention. CTLA-4 inhibitors reported to have higher risk for severe irAEs compared to PD1 or PD-L1 inhibitors in solid tumors, and the risk increases with the use of combination [65]. In a meta-analysis, CRC was found to have the highest incidence of ICI-induced irAEs at any grade, comprising 30.7% of other gastrointestinal cancers [66]. In KEYNOTE-177 trial, the reported irAEs were 31% with pembrolizumab and 15% with chemotherapy, and grade 3 or higher irAEs were 9% with pembrolizumab and 2% with chemotherapy. As expected, the combination of nivolumab and ipilimumab in CheckMate-8HW trial had higher irAEs (51% compared to 31% in the nivolumab arm); the rate of grade 3 or higher irAEs was of 16% dual ICI versus 6% with chemotherapy. In real-world clinical practice, ICI is well tolerated in patients with CRC in comparison to chemotherapy. Early detection of irAEs allows for early intervention and reduces the risk of morbidity and mortality.

The results of CheckMate 8HW trial positioned the ICI doublet of nivolumab with ipilimumab as a standard first-line therapy in dMMR/MSI-H advanced or mCRC alongside the single-agent embrolizumab. We prefer the dual ICI given the superior results in comparison to single-agent nivolumab. The decision between both regimens should incorporate availability, expected response, possible resistance, adverse-events profile and patient preference. In the presence of contraindications to ICI in dMMR/MSI-H mCRC (such as prior organ transplantation, pre-existing moderate-to-severe autoimmune disease, or a previous severe irAE), patients should be treated with standard chemotherapy with or without targeted therapy. On the rare occasion where a patient with dMMR/MSI mCRC did not receive ICI in the first line, we recommend the use of ICI in a subsequent treatment line on disease progression.

#### 3.1.4. Adjuvant Immunotherapy in dMMR/MSI-H Colon Cancer

The frequency of dMMR/MSI-H in stage 3 CRC is around 12% [67,68]; these malignancies have a favorable prognosis compared to pMMR/MSS CRC of the same stage. The benefit of adjuvant chemotherapy with a fluoropyrimidine in combination with oxaliplatin is well established in stage 3 colon cancer including dMMR/MSI subgroup [69]. The randomized phase 3 clinical trial ATOMIC investigated the addition of atezolizumab, an ICI that blocks PD-L1 receptors in tumor cells, to standard-of-care adjuvant chemotherapy in resected stage 3 dMMR colon cancer [70]. The study enrolled a total of 712 patients, who were randomized to receive either atezolizumab and FOLFOX chemotherapy (fluoropyrimidine and oxaliplatin) every two weeks for 12 cycles followed by atezolizumab alone for another 13 cycles or FOLFOX alone for 12 cycles. The results showed significant improvement in disease-free survival (DSF) in the atezolizumab group over chemotherapy alone, with 3-year DFS of 86.4% (95% CI 81.8–89.9) versus 76.6% (95% CI 71.3–81.0). There was 50% reduction in the risk of recurrence or death in the atezolizumab combination group compared to chemotherapy alone. The benefit of atezolizumab combination was reported in all subgroups including proximal colon cancer and high-risk stage 3 disease. Grade 3 adverse events were 71.7% in the atezolizumab group versus 62.1% in the chemotherapy alone group [70]. In the absence of contraindication to ICI, we recommend adding atezolizumab to FOLFOX adjuvant chemotherapy in resected stage 3 dMMR/MSI colon cancer.

The role of neoadjuvant ICI in dMMR/MSI colon cancer is being examined. NICHE-2 is a phase 2 single-arm clinical trial that showed promising results using neoadjuvant dual ICI with nivolumab and ipilimumab in locally advanced dMMR colon cancer [71]. The study enrolled 115 patients treated with one dose of ipilimumab and two doses of nivolumab before surgical resection. Pathological response was reported in 98% of patients (95% CI 94–100), with 67% (95% CI 58–76) having pathological complete response. At 26.2 months, no patients experienced disease recurrence, and thirty-seven patients had recorded follow up of more than 36 months with no disease recurrence. Despite the exciting results of NICHE-2 study, neoadjuvant ICI for resectable dMMR/MSI-H colorectal cancer remains experimental, and eligible patients should be offered clinical trial participation as a treatment option whenever possible.

#### 3.1.5. Practice-Changing Data in the Management of pMMR Metastatic Colorectal Cancer

The presence of BRAF mutation in mCRC is indicative of aggressive behavior and poor outcome. The use of a triplet chemotherapy regimen with anti-VGFR in this subgroup of patients with mCRC failed to show advantage in survival outcomes compared to doublet chemotherapy regimens with anti-VGFR [72]. The combination of anti-BRAF and anti-EGFR therapy is currently the treatment of choice in the second line setting for BRAF V600E mutant mCRC. The latest results of the randomized phase 3 clinical trial BREAKWATER confirmed that the combination of encorafenib (an anti-BRAF antibody) and cetuximab (an anti-EGFR antibody) with chemotherapy is a more effective first-line treatment option for mCRC with BRAF V600E mutations. The study compared the combination of encorafenib and cetuximab with modified FOLFOX chemotherapy against standard-of-care chemotherapy, which included doublet or triplet chemotherapy regimens with or without bevacizumab (an anti-VEGF antibody) [73]. The mPFS was 12.8 months with encorafenib and cetuximab combined with mFOLFOX, versus 7.1 months in the chemotherapy group (HR 0.53; 95% CI 0.41–0.68; *p* < 0.001). The combination encorafenib and cetuximab with mFOLFOX showed significantly better mOS (30.3 months, compared to 15.1 months in the chemotherapy group [HR 0.49; 95% CI 0.38– 0.68; *p* < 0.001]), and the final ORR was 65.7% (95% CI 59.4–71.4) in the combination group compared to 37.4% (95% CI 31.6–43.7) in the chemotherapy group [73]. Based on these results, we recommend the combination of encorafenib and cetuximab with FOLFOX chemotherapy as first-line therapy in pMMR/MSS mCRC with BRAF V600E mutations.

Around 21% of all BRAF mutant mCRC are also dMMR/MSI-H. Dual or single ICI is the most effective and preferred treatment option for dMMR/MSI-H mCRC, even in the setting of a coexistent BRAF mutation. The Breakwater trial did not include dMMR/MSI-H mCRC except patients who had BRAF V600E mutation and were ineligible for ICI. In patients experiencing disease progression on immune checkpoint inhibitors (ICIs), or in cases where ICIs are contraindicated. We recommend considering the combination of encorafenib and cetuximab with chemotherapy as a second-line or alternative treatment option for BRAF V600E dMMR/MSI mCRC.

## 4. Secondary Prevention of Colorectal Cancer

Question 1: What is the impact of a structured exercise program on outcomes in patients with resected high-risk stage II and III colorectal cancer?

• We recommend a structured exercise program as part of survivorship care in patients with resected high-risk stage II and III colorectal cancer who have received adjuvant chemotherapy [Level I].
• The intervention should include behavioral support, supervised physical activity, and long-term consultation with physical activity specialists [Level I].
  ○ Long-term consultation should include regular follow up by a physical activity consultant over a three-year period.

Question 2: What is the role of adjunctive ASA (Aspirin) therapy in patients with resected stage II–III colon cancer and I–III rectal cancer and PI3K pathway alterations?

• Adjunctive ASA (160 mg orally daily) for 3 years may be considered in patients with stage I–III rectal cancer and stage II–III colon cancer and somatic PI3K pathway alterations [Level I].
• There is a need to discuss the potential benefits and risks of ASA therapy with patients [Level I].

### 4.1. Summary of Evidence

#### 4.1.1. Structured Exercise in Resected Colorectal Cancer

Exercise is known to be beneficial for throughout the clinical course of most patients with cancer [74,75,76]. CO.21 CHALLENGE is a phase III trial that randomized patients with resected stage III or high-risk stage II colon cancer who completed adjuvant chemotherapy to undergo a structured exercise program or to receive health-education materials alone over a three-year period [77].

The health-education group received information about standard surveillance recommendations and general health information on nutrition and physical activity. The exercise group was provided with the same information, as well as an exercise guidebook designed for survivors of colon cancer and support from a certified physical activity consultant. The exercise program aimed to increase recreational physical activity from baseline to at least 10 Metabolic Equivalent Task (MET) hours per week within the first six months of the program, then (over the subsequent 2.5 years) remain at this level or further increase MET hours weekly at moderate intensity. The focus was on moderate-intensity (4 MET) exercise or higher, though exercise intensity as well as type and duration of aerobic exercise were left to the participant’s choice [77].

The first six months of the program (phase 1) included mandatory in-person participation in twelve behavioral support sessions which were combined with twelve supervised exercise sessions; an additional twelve exercise sessions were recommended, alternating weekly with the mandatory sessions. The second six months (phase 2) included twelve mandatory in-person or remote behavioral support sessions, delivered with supervised exercise with in-person attendance. The remaining two years consisted of 24 mandatory behavioral support sessions, attended either remotely or in-person (with supervised exercised sessions included with in-person attendance) [77].

Over a median follow-up time of 7.9 years, this trial demonstrated improved disease-free survival (HR 0.72; 95% CI 0.55–0.94, *p* = 0.02) and overall survival (HR 0.63; 95% CI 0.43–0.94) in the exercise group when compared to the health-education group. Exercise as an intervention is safe, with no increase in serious or life-threatening adverse events. Musculoskeletal adverse events were more frequent but mostly mild. It should be noted that, in the intervention arm of the CO.21 CHALLENGE study, a total of 48 behavioral support sessions with physical activity specialists were provided over the 3-year study period [77].

#### 4.1.2. Adjuvant ASA in Resected Colorectal Cancer

The phase III ALASCCA trial randomized patients with resected stage I to III rectal cancer and II to III colon cancer and a PIK3 pathway alteration (*PIK3CA* hotspot mutations in exon 9 or 20; Group A alterations), and moderate- or high-impact somatic variants in *PIK3CA*, *PIK3R1*, or *PTEN* (Group B alterations) to receive either ASA 160 mg orally once daily or placebo for three years [44]. Daily oral ASA 160 mg led to a significantly lower risk of colorectal cancer recurrence than placebo (HR 0.49; 95% CI 0.34–1.08, *p* = 0.04) in patients with PIK3CA hotspot mutations in exon 9 or 20, and it appeared to have a similar benefit among those with other relevant somatic alterations in PI3K pathway genes (Group B; *PIK3CA*, *PIK3R1*, or *PTEN*) [44].

In ALASCCA, severe adverse events occurred in 16.8% of ASA recipients, compared to 11.6% of placebo recipients. Of the fifty-two severe adverse events in the ASA, four were deemed by principal investigator assessment to be potentially related to aspirin use (allergic reaction, gastrointestinal bleed, spontaneous hematoma, and subarachnoid hemorrhage) [44]. Appropriate counseling about the risks and benefits of long-term ASA use remains important.

The result from the ALASCCA trial highlights the importance of upfront PIK3CA molecular testing. The role of ASA in all cases of resected colorectal cancer with PI3K pathway alterations (including those with resected oligometastatic disease) has not formally been supported by trial data at this time; due to ASA’s ease of administration, broad availability, reasonable safety profile, and clear mechanism of biomolecular efficacy in stage I to III colorectal cancer, ASA may similarly be considered in these patients.

## 5. Treatment of Hepatocellular Carcinoma (HCC)

Question 1: What are the current first-line treatment options for HCC patients being considered for systemic therapy in Canada?

• Appropriate first line treatment includes an ICI-based combination (atezolizumab + bevacizumab, durvalumab + tremelimumab, or nivolumab + ipilimumab) [Level I].
  ○ In the presence of contraindication to bevacizumab, doublet ICI should be offered.
• Oral TKI (tyrosine kinase inhibitor) (lenvatinib or sorafenib) can be used in the first-line treatment setting for patients with contraindication to ICI or based on patient’s preference [Level I].
• In case of disease progression or intolerance to ICI, second-line oral TKI can be used [Level II].
• Enrolment in clinical trials should be discussed with patients when available [Level III].

Question 2: Is there a role for adding systemic treatment to local therapy in intermediate-stage (stage B) HCC who are transplant-ineligible patients?

• Based on the immature data on OS, we cannot endorse the role of combination systemic treatment with local therapy in HCC [Level III].
  ○ Further maturing evidence from clinical trials is pending and this will need to be re-evaluated.

Question 3: Is there a role for adjuvant treatment after curative surgery or ablation in HCC with high risk of recurrence?

• Based on the current evidence, there is no role for adjuvant treatment with combination ICI plus VEGF inhibition, nor with an oral tyrosine kinase inhibitor [Level I].
  ○ Further evidence from clinical trials is pending and will need to be re-evaluated.

### 5.1. Summary of Evidence

#### 5.1.1. First-Line Systemic Therapies for Hepatocellular Carcinoma

The incidence of hepatocellular carcinoma (HCC) is increasing in Canada, with a majority of patients being diagnosed with advanced stage at presentation, precluding the chance for curative surgical resection or liver transplant [78]. The Barcelona Clinic Liver Cancer (BCLC) staging system is the most used staging system, and it has been adopted by multiple medical guidelines in staging and guiding the treatment recommendations in HCC. The 2022 version of the BCLC staging system integrates liver function status using the Child Pugh score, the Model for End-Stage Liver Disease (MELD) score, and the Albumin–Bilirubin (ALBI) score in addition to serum alpha-fetoprotein (AFP) and the patients’ performance status (using the Eastern Cooperative Oncology Group (ECOG) score) into the staging system to define the prognosis and treatment modality recommendations in HCC [79]. The latest 2025 update of the BCLC staging system, subsequently published following the conclusion of this consensus meeting, incorporates the CUSE framework, which stands for Complexity, Uncertainty, Subjectivity, and Emotion. This addition is intended to ensure that treatment decisions remain patient-centered, while maintaining consistent stage definitions and corresponding treatment options as in the 2022 version [80].

The BCLC staging system classifies HCC into five stages, ranging from very early stage (0) to advanced stage (C) and terminal stage (D), with further expansion of the (B) intermediate stage into three subgroups. The advanced stage (C) in BCLC is defined as HCC with portal vein invasion and/or extrahepatic spread, with preserved liver function and an ECOG performance status score of 1–2. Systemic treatment is recommended for advanced-stage (C) HCC and for intermediate-stage (B) HCC subgroups with diffuse, infiltrative, or bi-lobar liver involvement.

Historically, tyrosine kinases inhibitors (TKIs), sorafenib and lenvatinib, were the treatment of choice offered to patients with advanced-stage HCC. In 2020, ICIs entered the first-line systemic treatment landscape of HCC, pushing TKIs back to the second line. IMbrave-150 randomized phase 3 clinical trial was the first to show significant improvement in survival outcomes with ICI in the first-line treatment setting of advanced HCC [81]. The study enrolled patients with unresectable or advanced HCC who had not received any prior systemic therapy. Patients received either ICI-based combination of atezolizumab (anti-PD-L1) and bevacizumab (anti-VEGFR) or sorafenib. The mOS was longer in the atezolizumab-and-bevacizumab group compared to the sorafenib group (19.2 months versus 13.4 months [HR 0.66; 95% CI 0.52–0.85; descriptive *p* < 0.001]), as was the mPFS (6.9 months in the atezolizumab-and-bevacizumab group versus 4.3 months in the sorafenib group [HR 0.65; 95% CI 0.53–0.81; descriptive *p* < 0.001]). The confirmed ORR was 30% (95% CI 25–35) in the atezolizumab-and-bevacizumab group compared to 11% (95% CI 7–17) in the sorafenib group, with 8% of patients achieving complete clinical response with atezolizumab and bevacizumab. The reported grade 3 or 4 treatment-related adverse events were 43% and 46% in the atezolizumab + bevacizumab group and in the sorafenib group, respectively, and immune-related adverse events of grade 3 or more were seen in 25.8% of the atezolizumab + bevacizumab group [81].

The ICI combination of tremelimumab (an anti-CTLA-4 antibody) and durvalumab (an anti-PD-L1 antibody), known as the STRIDE regimen, has shown efficacy and improved outcomes in unresectable or advanced HCC in the first-line setting. This was evaluated in the phase 3 clinical trial HIMALAYA, which randomized patients to receive either combination ICI (with one dose of tremelimumab and durvalumab every four weeks), or single-agent durvalumab or sorafenib [82]. The primary end point was overall survival, comparing combination ICI (the STRIDE regimen) against sorafenib. The mOS (HR 0.76, *p* = 0.0008) for the STRIDE regimen was 16.43 months (95% CI 14.16–19.58) versus 13.77 months (95% CI 12.25–16.13) for sorafenib. The survival rates at 36, 48, and 60 months were 30.7%, 25.2%, and 19.6%, respectively, in the STRIDE group compared to 19.8%, 15.1%, and 9.6%, respectively, in the sorafenib group. There was no significant difference in mPFS between the two groups (4.9 months, HR 0.90; 95% CI 0.77–1.05). At the time of the final analysis, the ORR was 51.5% in the STRIDE group compared to 15.6% in the sorafenib group, with 11.7% of patients on STRIDE having complete clinical response [83]. Grade 3 or 4 treatment-related adverse events were 25.8% in the STRIDE group and 36.9% in the sorafenib group, and the immune-related adverse events of grade 3 or more were reported at 14.1% with STRIDE. Additionally, the study showed that single-agent durvalumab is non-inferior to sorafenib (HR 0.86; 95.67% CI 0.73–1.03; noninferiority margin, 1.08) [83].

Recently, the CheckMate 9DW randomized phase 3 clinical trial showed a survival benefit in the first-line setting with dual ICI nivolumab and ipilimumab over investigator choice of sorafenib or lenvatinib in unresectable or advanced HCC [84]. The mOS was 23.7 months in the dual ICI compared to 20.6 months in the TKI group (HR 0.79; 95% CI 0.65–0.96), and the 36 months overall survival was 38% (95% CI 32–43) in the dual ICI compared to 24% (95% CI 19–30) in the TKI group. The mPFS was comparable in both arms; however, the 24-month progression survival rate was 28% (95% CI 23–34) in the dual ICI group versus 12% (95% CI 8–17) in the TKI group. The ORR favored the ICI doublet at 36% (95% CI 31–42) versus 13% (95% CI 10–17) in the TKI group, with complete clinical response seen in 7% on the dual ICI group. Grade 3 or 4 treatment-related adverse event rates were 41% in the dual ICI group and 42% in the TKI group, while the immune-related adverse events of any grade were reported as 58% with the dual ICI group [84].

We recommend ICI-based combination for the first-line treatment of advanced or unresectable HCC. Based on the evidence outlined above, options include atezolizumab and bevacizumab, tremelimumab and durvalumab (STRIDE regimen), and nivolumab and ipilimumab. The decision between these regimens should consider history of uncontrolled esophageal varices, bleeding and thrombosis risks, liver functional status, patient’s medical comorbidities, performance status, the treatment’s adverse event profile, and patient’s preference. In the presence of contraindication to ICI, we recommend using lenvatinib or sorafenib in the first line for unresectable advanced stage HCC; lenvatinib is generally preferred because of its superior tolerability. Radiotherapy is an option that can be considered for symptomatic liver lesions, bone metastasis, or for hemostasis. Palliative/best supportive care should be discussed with all patients with advanced-stage (C) and terminal-stage (D) HCC.

#### 5.1.2. Addition of Systemic Therapy to Local Therapy in Intermediate-Stage (Stage B) Transplant-Ineligible Patients with Hepatocellular Carcinoma

The intermediate stage (stage B) in BCLC covers a heterogeneous group of HCC with only small number of patients reach liver transplant after successful downstaging. Trans-arterial chemoembolization (TACE) has been the preferred modality for this group, either before liver transplant or for local control when curative treatment cannot be pursued. Despite the efficacy of TACE in providing disease control and delaying progression in HCC, half of the patients will have disease recurrence within 6 months, with the majority of these being local recurrences [85]. Locally advanced HCC, multifocal disease, tumors larger than 5 cm, underlying liver dysfunction, and patient’s performance status all are factors confounding the efficacy of TACE. Other locoregional therapies can be used according to the center expertise and/or availability.

TACE allows delivery of chemotherapy directly into the tumor vasculature, which subsequently induces ischemia and tumor necrosis. This has led to the consideration of potential synergistic effects between TACE and systemic therapy. Earlier studies investigated combining sorafenib with TACE in unresectable HCC, with conflicting results and no statistically significant survival benefits over TACE alone in phase 3 studies [86,87]. Recently, three randomized phase 3 clinical trials showed promising results with combined treatment modalities with TACE and ICI-based therapy in nonmetastatic but unresectable HCC. LEAP-012, a randomized multicenter phase 3 clinical trial, showed improvement in mPFS with pembrolizumab and lenvatinib combined with TACE for 14·6 months (95% CI 12·6–16·7) versus 10 months (95% CI 8.1–12.2) with TACE and placebo (HR 0·66, one-sided *p* = 0·0002), with signs of improvement in overall survival rate [88]. In an EMERALD-1 phase 3 clinical trial, randomized patients were to receive either durvalumab and bevacizumab with TACE, durvalumab alone with TACE, or TACE only [89]. The combination of durvalumab and bevacizumab with TACE demonstrated an improvement in mPFS to 15 months (HR 0.77; 95% CI 11.1–18.9) compared to 8·2 months (HR 0·77; 95% CI 6.9–11.1) in the placebo with TACE arm. Comparable results were reported in the TALENTACE phase 3 randomized clinical trial, which showed an improvement in mPFS with the combination of atezolizumab and bevacizumab with TACE to 10.32 months, versus 6.37 months with TACE alone (HR 0.64; 95% CI 0.50–0.82, *p* < 0.001) [90]. In their initial analyses, these three clinical trials showed improved overall response rates with the addition of ICI-based combination therapy to TACE compared to TACE alone; however, overall survival outcomes are pending.

Current evidence suggests a potential role for combining systemic therapy (ICI and anti-VEGF agents or tyrosine kinase inhibitors) with locoregional treatments in patients with locally advanced nonmetastatic HCC who are not eligible for surgery; however, further robust data are necessary to substantiate this approach. Patient enrolment in clinical trials should be encouraged whenever feasible.

#### 5.1.3. Role of Adjuvant Treatment Following Curative Surgery or Ablation in Hepatocellular Carcinoma at High Risk of Recurrence

Liver transplant and surgical resection are the primary curative therapies in early-stage HCC. The recurrence rate is higher after liver resection compared to after liver transplantation, with an estimated 5-year recurrence rate of up to 70% and resection compared to 8–20% after liver transplantation [91]. Disease recurrence within the first two years after hepatectomy are considered to be early recurrences and linked to the aggressiveness of HCC, while late recurrences are more commonly linked to underlying liver cirrhosis. HCC with high risk of recurrence is characterized by the presence of multinodular tumors (up to 3 foci)—with the largest measuring >5 cm, the presence of microvascular invasion, or segmental portal vein involvement (VP1 or VP2)—or multinodular tumors with 4 or more tumors each measuring less than 5 cm. Additional features that increase the risk of recurrence include a poorly differentiated histology and high serum levels of alpha-fetoprotein.

Numerous adjuvant therapies have been investigated in hopes of decreasing the risk of postoperative recurrences. Although several approaches, with either locoregional therapy or systemic treatment, have demonstrated efficacy in lowering recurrence rates and enhancing overall survival, a unified consensus regarding adjuvant treatment is not currently established within international HCC guidelines. The phase 3 randomized clinical trial STORM demonstrated that adjuvant sorafenib did not confer any survival advantage in HCC after curative resection or ablation. The recurrence-free survival was 33.3 months in the sorafenib group versus 33.7 months in the placebo group (HR 0.940; 95% CI 0.780–1.134) [92]. ICI-based combinations have also been explored in the adjuvant setting; IMbrave-050 is a randomized phase 3 clinical trial that investigated adjuvant atezolizumab and bevacizumab compared to active surveillance after curative resection or ablation in high-risk HCC [93]. The median recurrence-free survival (RFS) was not reached at the time of first interim analysis; the one-year RFS rate was of 78% in the atezolizumab and bevacizumab group compared to 65% in the surveillance group (HR 0.72; 95% CI: 0.56–0.93, *p* = 0.01) [93]. Although these results are promising, longer follow-up time is needed to confirm survival benefits and efficacy. Toxicity is another factor to be considered with adjuvant ICI; grade 3 or 4 adverse events in IMbrave-050 were 41% in the atezolizumab-and-bevacizumab group compared to 13% in the active surveillance group. Additional clinical trials evaluating the efficacy of adjuvant ICI following curative therapy for hepatocellular carcinoma are currently in progress. The current evidence does not support the use of adjuvant therapy in hepatocellular carcinoma (HCC) following curative-intent surgical resection or ablation. Patient enrolment in clinical trials should be encouraged whenever feasible.

## Data Availability

No new data were created or analyzed in this study. Data sharing is not applicable to this article.

## References

[1] Agarwal S., Bell M.G., Dhaliwal L., Codipilly D.C., Dierkhising R.A., Lansing R., Gibbons E.E., Leggett C.L., Kisiel J.B., Iyer P.G. (2024). Correction: Population Based Time Trends in the Epidemiology and Mortality of Gastroesophageal Junction and Esophageal Adenocarcinoma. Dig. Dis. Sci..

[2] Bang Y.J., Van Cutsem E., Feyereislova A., Chung H.C., Shen L., Sawaki A., Lordick F., Ohtsu A., Omuro Y., Satoh T. (2010). Trastuzumab in combination with chemotherapy versus chemotherapy alone for treatment of HER2-positive advanced gastric or gastro-oesophageal junction cancer (ToGA): A phase 3, open-label, randomised controlled trial. Lancet.

[3] Janjigian Y.Y., Kawazoe A., Yañez P., Li N., Lonardi S., Kolesnik O., Barajas O., Bai Y., Shen L., Tang Y. (2021). The KEYNOTE-811 trial of dual PD-1 and HER2 blockade in HER2-positive gastric cancer. Nature.

[4] Janjigian Y.Y., Shitara K., Moehler M., Garrido M., Salman P., Shen L., Wyrwicz L., Yamaguchi K., Skoczylas T., Campos Bragagnoli A. (2021). First-line nivolumab plus chemotherapy versus chemotherapy alone for advanced gastric, gastro-oesophageal junction, and oesophageal adenocarcinoma (CheckMate 649): A randomised, open-label, phase 3 trial. Lancet.

[5] Rha S.Y., Oh D.Y., Yañez P., Bai Y., Ryu M.H., Lee J., Rivera F., Alves G.V., Garrido M., Shiu K.K. (2023). Pembrolizumab plus chemotherapy versus placebo plus chemotherapy for HER2-negative advanced gastric cancer (KEYNOTE-859): A multicentre, randomised, double-blind, phase 3 trial. Lancet Oncol..

[6] (2025). Imfinzi-Based Regimen Reduced the Risk of Death by 22% in Early Gastric Cancer vs. Chemotherapy Alone in MATTERHORN Phase III Trial. https://www.astrazeneca.com/content/astraz/media-centre/press-releases/2025/imfinzi-based-regimen-reduced-the-risk-of-death-by-22-percent-in-early-gastric-cancer-vs-chemotherapy-alone-in-matterhorn-phase-iii-trial.html.

[7] Shah M.A., Shitara K., Ajani J.A., Bang Y.J., Enzinger P., Ilson D., Lordick F., Van Cutsem E., Gallego Plazas J., Huang J. (2023). Zolbetuximab plus CAPOX in CLDN18.2-positive gastric or gastroesophageal junction adenocarcinoma: The randomized, phase 3 GLOW trial. Nat. Med..

[8] Shitara K., Lordick F., Bang Y.J., Enzinger P., Ilson D., Shah M.A., Van Cutsem E., Xu R.H., Aprile G., Xu J. (2023). Zolbetuximab plus mFOLFOX6 in patients with CLDN18.2-positive, HER2-negative, untreated, locally advanced unresectable or metastatic gastric or gastro-oesophageal junction adenocarcinoma (SPOTLIGHT): A multicentre, randomised, double-blind, phase 3 trial. Lancet.

[9] Chao J., Fuchs C.S., Shitara K., Tabernero J., Muro K., Van Cutsem E., Bang Y.J., De Vita F., Landers G., Yen C.J. (2021). Assessment of Pembrolizumab Therapy for the Treatment of Microsatellite Instability–High Gastric or Gastroesophageal Junction Cancer Among Patients in the KEYNOTE-059, KEYNOTE-061, and KEYNOTE-062 Clinical Trials. JAMA Oncol..

[10] Shitara K., Van Cutsem E., Bang Y.J., Fuchs C., Wyrwicz L., Lee K.W., Kudaba I., Garrido M., Chung H.C., Lee J. (2020). Efficacy and Safety of Pembrolizumab or Pembrolizumab Plus Chemotherapy vs Chemotherapy Alone for Patients with First-line, Advanced Gastric Cancer: The KEYNOTE-062 Phase 3 Randomized Clinical Trial. JAMA Oncol..

[11] André T., Tougeron D., Piessen G., de la Fouchardière C., Louvet C., Adenis A., Jary M., Tournigand C., Aparicio T., Desrame J. (2023). Neoadjuvant Nivolumab Plus Ipilimumab and Adjuvant Nivolumab in Localized Deficient Mismatch Repair/Microsatellite Instability-High Gastric or Esophagogastric Junction Adenocarcinoma: The GERCOR NEONIPIGA Phase II Study. J. Clin. Oncol..

[12] Cunningham D., Allum W.H., Stenning S.P., Thompson J.N., Velde C.J.H.V., de Nicolson M., Scarffe J.H., Lofts F.J., Falk S.J., Iveson T.J. (2006). Perioperative Chemotherapy versus Surgery Alone for Resectable Gastroesophageal Cancer. N. Engl. J. Med..

[13] Ychou M., Boige V., Pignon J.P., Conroy T., Bouché O., Lebreton G., Ducourtieux M., Bedenne L., Fabre J.M., Saint-Aubert B. (2011). Perioperative Chemotherapy Compared with Surgery Alone for Resectable Gastroesophageal Adenocarcinoma: An FNCLCC and FFCD Multicenter Phase III Trial. J. Clin. Oncol..

[14] Van Hagen P., Hulshof M.C.C.M., Van Lanschot J.J.B., Steyerberg E.W., Henegouwen M.I.V.B., Wijnhoven B.P.L., Richel D.J., Nieuwenhuijzen G.A.P., Hospers G.A.P., Bonenkamp J.J. (2012). Preoperative Chemoradiotherapy for Esophageal or Junctional Cancer. N. Engl. J. Med..

[15] Al-Batran S.E., Homann N., Pauligk C., Goetze T.O., Meiler J., Kasper S., Kopp H.G., Mayer F., Haag G.M., Luley K. (2019). Perioperative chemotherapy with fluorouracil plus leucovorin, oxaliplatin, and docetaxel versus fluorouracil or capecitabine plus cisplatin and epirubicin for locally advanced, resectable gastric or gastro-oesophageal junction adenocarcinoma (FLOT4): A randomised, phase 2/3 trial. Lancet.

[16] Reynolds J.V., Preston S.R., O’Neill B., Lowery M.A., Baeksgaard L., Crosby T., Cunningham M., Cuffe S., Griffiths G.O., Parker I. (2023). Trimodality therapy versus perioperative chemotherapy in the management of locally advanced adenocarcinoma of the oesophagus and oesophagogastric junction (Neo-AEGIS): An open-label, randomised, phase 3 trial. Lancet Gastroenterol. Hepatol..

[17] Hoeppner J., Brunner T., Schmoor C., Bronsert P., Kulemann B., Claus R., Utzolino S., Izbicki J.R., Gockel I., Gerdes B. (2025). Perioperative Chemotherapy or Preoperative Chemoradiotherapy in Esophageal Cancer. N. Engl. J. Med..

[18] Leong T., Smithers B.M., Michael M., Haustermans K., Wong R., Gebski V., O’Connell R.L., Zalcberg J., Boussioutas A., Findlay M. (2024). Preoperative Chemoradiotherapy for Resectable Gastric Cancer. N. Engl. J. Med..

[19] Kelly R.J., Ajani J.A., Kuzdzal J., Zander T., Van Cutsem E., Piessen G., Mendez G., Feliciano J., Motoyama S., Lièvre A. (2021). Adjuvant Nivolumab in Resected Esophageal or Gastroesophageal Junction Cancer. N. Engl. J. Med..

[20] Kelly R.J., Ajani J.A., Kuzdzal J., Zander T., Cutsem E.V., Piessen G., Mendez G., Feliciano J.L., Motoyama S., Lievre A. (2025). Adjuvant nivolumab in resected esophageal or gastroesophageal junction cancer (EC/GEJC) following neoadjuvant chemoradiotherapy (CRT): First results of overall survival (OS) from CheckMate 577. J. Clin. Oncol..

[21] Shitara K., Rha S.Y., Wyrwicz L.S., Oshima T., Karaseva N., Osipov M., Yasui H., Yabusaki H., Afanasyev S., Park Y.K. (2024). Neoadjuvant and adjuvant pembrolizumab plus chemotherapy in locally advanced gastric or gastro-oesophageal cancer (KEYNOTE-585): An interim analysis of the multicentre, double-blind, randomised phase 3 study. Lancet Oncol..

[22] Shitara K., Rha S.Y., Wyrwicz L., Oshima T., Karaseva N., Osipov M., Yasui H., Yabusaki H., Afanasyev S., Park Y.K. (2025). Pembrolizumab Plus Chemotherapy Versus Chemotherapy as Perioperative Therapy in Locally Advanced Gastric and Gastroesophageal Junction Cancer: Final Analysis of the Randomized, Phase III KEYNOTE-585 Study. J. Clin. Oncol..

[23] Janjigian Y.Y., Al-Batran S.E., Wainberg Z.A., Muro K., Molena D., Cutsem E.V., Hyung W.J., Wyrwicz L., Oh D.Y., Omori T. (2025). Perioperative Durvalumab in Gastric and Gastroesophageal Junction Cancer. N. Engl. J. Med..

[24] Koopman M., Kortman G.A.M., Mekenkamp L., Ligtenberg M.J.L., Hoogerbrugge N., Antonini N.F., Punt C.J.A., van Krieken J.H.J.M. (2009). Deficient mismatch repair system in patients with sporadic advanced colorectal cancer. Br. J. Cancer.

[25] Popat S., Hubner R., Houlston R.S. (2005). Systematic review of microsatellite instability and colorectal cancer prognosis. J. Clin. Oncol..

[26] Vaughn C.P., Zobell S.D., Furtado L.V., Baker C.L., Samowitz W.S. (2011). Frequency of KRAS, BRAF, and NRAS mutations in colorectal cancer. Genes Chromosomes Cancer.

[27] Innocenti F., Ou F.S., Qu X., Zemla T.J., Niedzwiecki D., Tam R., Mahajan S., Goldberg R.M., Bertagnolli M.M., Blanke C.D. (2019). Mutational Analysis of Patients with Colorectal Cancer in CALGB/SWOG 80405 Identifies New Roles of Microsatellite Instability and Tumor Mutational Burden for Patient Outcome. J. Clin. Oncol..

[28] Cutsem E.V., Hitre E., Makhson A., Lim R., Roh J.K., Stroh C., Schlichting M., Rougier P. (2009). Cetuximab and Chemotherapy as Initial Treatment for Metastatic Colorectal Cancer. N. Engl. J. Med..

[29] Van Cutsem E., Lenz H.J., Köhne C.H., Heinemann V., Tejpar S., Melezínek I., Beier F., Stroh C., Rougier P., van Krieken J.H. (2015). Fluorouracil, leucovorin, and irinotecan plus cetuximab treatment and RAS mutations in colorectal cancer. J. Clin. Oncol..

[30] Douillard J.Y., Siena S., Cassidy J., Tabernero J., Burkes R., Barugel M., Humblet Y., Bodoky G., Cunningham D., Jassem J. (2014). Final results from PRIME: Randomized phase III study of panitumumab with FOLFOX4 for first-line treatment of metastatic colorectal cancer. Ann. Oncol..

[31] Fakih M.G., Kopetz S., Kuboki Y., Kim T.W., Munster P.N., Krauss J.C., Falchook G.S., Han S.W., Heinemann V., Muro K. (2022). Sotorasib for previously treated colorectal cancers with KRASG12C mutation (CodeBreaK100): A prespecified analysis of a single-arm, phase 2 trial. Lancet Oncol..

[32] Yaeger R., Weiss J., Pelster M.S., Spira A.I., Barve M., Ou S.H.I., Leal T.A., Bekaii-Saab T.S., Paweletz C.P., Heavey G.A. (2023). Adagrasib with or without Cetuximab in Colorectal Cancer with Mutated KRAS G12C. N. Engl. J. Med..

[33] Fakih M.G., Salvatore L., Esaki T., Modest D.P., Lopez-Bravo D.P., Taieb J., Karamouzis M.V., Ruiz-Garcia E., Kim T.W., Kuboki Y. (2023). Sotorasib plus Panitumumab in Refractory Colorectal Cancer with Mutated KRAS G12C. N. Engl. J. Med..

[34] de Langen A.J., Johnson M.L., Mazieres J., Dingemans A.M.C., Mountzios G., Pless M., Wolf J., Schuler M., Lena H., Skoulidis F. (2023). Sotorasib versus docetaxel for previously treated non-small-cell lung cancer with KRASG12C mutation: A randomised, open-label, phase 3 trial. Lancet.

[35] Barlesi F., Yao W., Duruisseaux M., Doucet L., Martínez A.A., Gregorc V., Juan-Vidal O., Lu S., De Bondt C., de Marinis F. (2025). Adagrasib versus docetaxel in KRASG12C-mutated non-small-cell lung cancer (KRYSTAL-12): A randomised, open-label, phase 3 trial. Lancet.

[36] Nassar A.H., Adib E., Kwiatkowski D.J. (2021). Distribution of KRAS G12C Somatic Mutations across Race, Sex, and Cancer Type. N. Engl. J. Med..

[37] Clarke C.N., Kopetz E.S. (2015). BRAF mutant colorectal cancer as a distinct subset of colorectal cancer: Clinical characteristics, clinical behavior, and response to targeted therapies. J. Gastrointest. Oncol..

[38] Jones J.C., Renfro L.A., Al-Shamsi H.O., Schrock A.B., Rankin A., Zhang B.Y., Kasi P.M., Voss J.S., Leal A.D., Sun J. (2017). Non-V600 BRAF Mutations Define a Clinically Distinct Molecular Subtype of Metastatic Colorectal Cancer. J. Clin. Oncol..

[39] Sinicrope F.A., Mahoney M.R., Yoon H.H., Smyrk T.C., Thibodeau S.N., Goldberg R.M., Nelson G.D., Sargent D.J., Alberts S.R. (2015). Analysis of Molecular Markers by Anatomic Tumor Site in Stage III Colon Carcinomas from Adjuvant Chemotherapy Trial NCCTG N0147 (Alliance). Clin. Cancer Res..

[40] Souglakos J., Philips J., Wang R., Marwah S., Silver M., Tzardi M., Silver J., Ogino S., Hooshmand S., Kwak E. (2009). Prognostic and predictive value of common mutations for treatment response and survival in patients with metastatic colorectal cancer. Br. J. Cancer.

[41] Di Nicolantonio F., Martini M., Molinari F., Sartore-Bianchi A., Arena S., Saletti P., De Dosso S., Mazzucchelli L., Frattini M., Siena S. (2008). Wild-type BRAF is required for response to panitumumab or cetuximab in metastatic colorectal cancer. J. Clin. Oncol..

[42] Kopetz S., Grothey A., Yaeger R., Cutsem E.V., Desai J., Yoshino T., Wasan H., Ciardiello F., Loupakis F., Hong Y.S. (2019). Encorafenib, Binimetinib, and Cetuximab in BRAF V600E–Mutated Colorectal Cancer. N. Engl. J. Med..

[43] Rosty C., Young J.P., Walsh M.D., Clendenning M., Sanderson K., Walters R.J., Parry S., Jenkins M.A., Win A.K., Southey M.C. (2013). PIK3CA Activating Mutation in Colorectal Carcinoma: Associations with Molecular Features and Survival. PLoS ONE.

[44] Martling A., Hed Myrberg I., Nilbert M., Grönberg H., Granath F., Eklund M., Öresland T., Iversen L.H., Haapamäki C., Janson M. (2025). Low-Dose Aspirin for PI3K-Altered Localized Colorectal Cancer. N. Engl. J. Med..

[45] Ross J.S., Fakih M., Ali S.M., Elvin J.A., Schrock A.B., Suh J., Vergilio J.A., Ramkissoon S., Severson E., Daniel S. (2018). Targeting HER2 in colorectal cancer: The landscape of amplification and short variant mutations in ERBB2 and ERBB3. Cancer.

[46] Yonesaka K., Zejnullahu K., Okamoto I., Satoh T., Cappuzzo F., Souglakos J., Ercan D., Rogers A., Roncalli M., Takeda M. (2011). Activation of ERBB2 signaling causes resistance to the EGFR-directed therapeutic antibody cetuximab. Sci. Transl. Med..

[47] Sartore-Bianchi A., Trusolino L., Martino C., Bencardino K., Lonardi S., Bergamo F., Zagonel V., Leone F., Depetris I., Martinelli E. (2016). Dual-targeted therapy with trastuzumab and lapatinib in treatment-refractory, KRAS codon 12/13 wild-type, HER2-positive metastatic colorectal cancer (HERACLES): A proof-of-concept, multicentre, open-label, phase 2 trial. Lancet Oncol..

[48] Meric-Bernstam F., Hurwitz H., Raghav K.P.S., McWilliams R.R., Fakih M., VanderWalde A., Swanton C., Kurzrock R., Burris H., Sweeney C. (2019). Pertuzumab plus trastuzumab for HER2-amplified metastatic colorectal cancer (MyPathway): An updated report from a multicentre, open-label, phase 2a, multiple basket study. Lancet Oncol..

[49] Strickler J.H., Cercek A., Siena S., André T., Ng K., Van Cutsem E., Wu C., Paulson A.S., Hubbard J.M., Coveler A.L. (2023). Tucatinib plus trastuzumab for chemotherapy-refractory, HER2-positive, RAS wild-type unresectable or metastatic colorectal cancer (MOUNTAINEER): A multicentre, open-label, phase 2 study. Lancet Oncol..

[50] Yoshino T., Di Bartolomeo M., Raghav K., Masuishi T., Loupakis F., Kawakami H., Yamaguchi K., Nishina T., Wainberg Z., Elez E. (2023). Final results of DESTINY-CRC01 investigating trastuzumab deruxtecan in patients with HER2-expressing metastatic colorectal cancer. Nat. Commun..

[51] Raghav K., Siena S., Takashima A., Kato T., Van den Eynde M., Pietrantonio F., Komatsu Y., Kawakami H., Peeters M., Andre T. (2024). Trastuzumab deruxtecan in patients with HER2-positive advanced colorectal cancer (DESTINY-CRC02): Primary results from a multicentre, randomised, phase 2 trial. Lancet Oncol..

[52] Strickler J.H., Bekaii-Saab T., Cercek A., Heinemann V., Nakamura Y., Raghav K., Siena S., Tabernero J., Van Cutsem E., Yoshino T. (2025). MOUNTAINEER-03 phase III study design: First-line mFOLFOX6 + tucatinib + trastuzumab for HER2+ metastatic colorectal cancer. Future Oncol..

[53] Cancer Genome Atlas Network (2012). Comprehensive molecular characterization of human colon and rectal cancer. Nature.

[54] Loree J.M., Bailey A.M., Johnson A.M., Yu Y., Wu W., Bristow C.A., Davis J.S., Shaw K.R., Broaddus R., Banks K.C. (2018). Molecular Landscape of ERBB2/ERBB3 Mutated Colorectal Cancer. J. Natl. Cancer Inst..

[55] Lasota J., Chłopek M., Lamoureux J., Christiansen J., Kowalik A., Wasąg B., Felisiak-Gołąbek A., Agaimy A., Biernat W., Canzonieri V. (2020). Colonic Adenocarcinomas Harboring NTRK Fusion Genes: A Clinicopathologic and Molecular Genetic Study of 16 Cases and Review of the Literature. Am. J. Surg. Pathol..

[56] Svrcek M., Cayre A., Samaille T., Colle R., Mas L., Bourgoin P., Guillerm E., Cohen R., Penault-Llorca F., André T. (2024). High prevalence of NTRK fusions in sporadic dMMR/MSI mCRC RAS/RAF wild-type: An opportunity for a post-immune checkpoint inhibitors progression rescue strategy. ESMO Gastrointest. Oncol..

[57] Hong D.S., DuBois S.G., Kummar S., Farago A.F., Albert C.M., Rohrberg K.S., van Tilburg C.M., Nagasubramanian R., Berlin J.D., Federman N. (2020). Larotrectinib in patients with TRK fusion-positive solid tumours: A pooled analysis of three phase 1/2 clinical trials. Lancet Oncol..

[58] Qi C., Shen L., Andre T., Chung H.C., Cannon T.L., Garralda E., Italiano A., Rieke D.T., Liu T., Burcoveanu D.I. (2025). Efficacy and safety of larotrectinib in patients with TRK fusion gastrointestinal cancer. Eur. J. Cancer.

[59] Overman M.J., McDermott R., Leach J.L., Lonardi S., Lenz H.J., Morse M.A., Desai J., Hill A., Axelson M., Moss R.A. (2017). Nivolumab in patients with metastatic DNA mismatch repair-deficient or microsatellite instability-high colorectal cancer (CheckMate 142): An open-label, multicentre, phase 2 study. Lancet Oncol..

[60] André T., Lonardi S., Wong K.Y.M., Lenz H.J., Gelsomino F., Aglietta M., Morse M.A., Van Cutsem E., McDermott R., Hill A. (2022). Nivolumab plus low-dose ipilimumab in previously treated patients with microsatellite instability-high/mismatch repair-deficient metastatic colorectal cancer: 4-year follow-up from CheckMate 142. Ann. Oncol..

[61] Le D.T., Diaz L.A., Kim T.W., Van Cutsem E., Geva R., Jäger D., Hara H., Burge M., O’Neil B.H., Kavan P. (2023). Pembrolizumab for previously treated, microsatellite instability–high/mismatch repair–deficient advanced colorectal cancer: Final analysis of KEYNOTE-164. Eur. J. Cancer.

[62] Diaz L.A., Shiu K.K., Kim T.W., Jensen B.V., Jensen L.H., Punt C., Smith D., Garcia-Carbonero R., Benavides M., Gibbs P. (2022). Pembrolizumab versus chemotherapy for microsatellite instability-high or mismatch repair-deficient metastatic colorectal cancer (KEYNOTE-177): Final analysis of a randomised, open-label, phase 3 study. Lancet Oncol..

[63] Andre T., Elez E., Van Cutsem E., Jensen L.H., Bennouna J., Mendez G., Schenker M., de la Fouchardiere C., Limon M.L., Yoshino T. (2024). Nivolumab plus Ipilimumab in Microsatellite-Instability-High Metastatic Colorectal Cancer. N. Engl. J. Med..

[64] André T., Elez E., Lenz H.J., Jensen L.H., Touchefeu Y., Van Cutsem E., Garcia-Carbonero R., Tougeron D., Mendez G.A., Schenker M. (2025). Nivolumab plus ipilimumab versus nivolumab in microsatellite instability-high metastatic colorectal cancer (CheckMate 8HW): A randomised, open-label, phase 3 trial. Lancet.

[65] Marin-Acevedo J.A., Chirila R.M., Dronca R.S. (2019). Immune Checkpoint Inhibitor Toxicities. Mayo Clin. Proc..

[66] Kou L., Wen Q., Xie X., Chen X., Li J., Li Y. (2022). Adverse events of immune checkpoint inhibitors for patients with digestive system cancers: A systematic review and meta-analysis. Front. Immunol..

[67] Mulet-Margalef N., Linares J., Badia-Ramentol J., Jimeno M., Sanz Monte C., Manzano Mozo J.L., Calon A. (2023). Challenges and Therapeutic Opportunities in the dMMR/MSI-H Colorectal Cancer Landscape. Cancers.

[68] ATOMIC: Atezolizumab + FOLFOX Significantly Improves DFS, Reduces Recurrence Risk in Stage III dMMR Colon Cancer. https://dailynews.ascopubs.org/do/10.1200/adn.25.300195/full/.

[69] Tougeron D., Mouillet G., Trouilloud I., Lecomte T., Coriat R., Aparicio T., Des Guetz G., Lécaille C., Artru P., Sickersen G. (2016). Efficacy of Adjuvant Chemotherapy in Colon Cancer With Microsatellite Instability: A Large Multicenter AGEO Study. J. Natl. Cancer Inst..

[70] Sinicrope F.A., Ou F.S., Arnold D., Peters W., Behrens R.J., Lieu C.H., Matin K., Cohen D.J., Potter S.L., Frankel W.L. (2025). Randomized trial of standard chemotherapy alone or combined with atezolizumab as adjuvant therapy for patients with stage III deficient DNA mismatch repair (dMMR) colon cancer (Alliance A021502; ATOMIC). J. Clin. Oncol..

[71] Chalabi M., Verschoor Y.L., Tan P.B., Balduzzi S., Van Lent A.U., Grootscholten C., Dokter S., Büller N.V., Grotenhuis B.A., Kuhlmann K. (2024). Neoadjuvant Immunotherapy in Locally Advanced Mismatch Repair-Deficient Colon Cancer. N. Engl. J. Med..

[72] Cremolini C., Antoniotti C., Stein A., Bendell J., Gruenberger T., Rossini D., Masi G., Ongaro E., Hurwitz H., Falcone A. (2020). Individual Patient Data Meta-Analysis of FOLFOXIRI Plus Bevacizumab Versus Doublets Plus Bevacizumab as Initial Therapy of Unresectable Metastatic Colorectal Cancer. J. Clin. Oncol..

[73] Elez E., Yoshino T., Shen L., Lonardi S., Van Cutsem E., Eng C., Kim T.W., Wasan H.S., Desai J., Ciardiello F. (2025). Encorafenib, Cetuximab, and mFOLFOX6 in BRAF-Mutated Colorectal Cancer. N. Engl. J. Med..

[74] Knols R., Aaronson N.K., Uebelhart D., Fransen J., Aufdemkampe G. (2005). Physical Exercise in Cancer Patients During and After Medical Treatment: A Systematic Review of Randomized and Controlled Clinical Trials. J. Clin. Oncol..

[75] Spence R.R., Heesch K.C., Brown W.J. (2010). Exercise and cancer rehabilitation: A systematic review. Cancer Treat. Rev..

[76] D’Ascenzi F., Anselmi F., Fiorentini C., Mannucci R., Bonifazi M., Mondillo S. (2021). The benefits of exercise in cancer patients and the criteria for exercise prescription in cardio-oncology. Eur. J. Prev. Cardiol..

[77] Courneya K.S., Vardy J.L., O’Callaghan C.J., Gill S., Friedenreich C.M., Wong R.K.S., Dhillon H.M., Coyle V., Chua N.S., Jonker D.J. (2025). Structured Exercise after Adjuvant Chemotherapy for Colon Cancer. N. Engl. J. Med..

[78] Canadian Cancer Statistics|Canadian Cancer Society. https://cancer.ca/en/research/cancer-statistics/canadian-cancer-statistics.

[79] Reig M., Forner A., Rimola J., Ferrer-Fàbrega J., Burrel M., Garcia-Criado Á., Kelley R.K., Galle P.R., Mazzaferro V., Salem R. (2022). BCLC strategy for prognosis prediction and treatment recommendation: The 2022 update. J. Hepatol..

[80] Reig M., Sanduzzi-Zamparelli M., Forner A., Rimola J., Ferrer-Fàbrega J., Burrel M., Garcia-Criado Á., Díaz A., Llarch N., Iserte G. (2025). BCLC strategy for prognosis prediction and treatment recommendations: The 2025 update. J. Hepatol..

[81] Cheng A.L., Qin S., Ikeda M., Galle P.R., Ducreux M., Kim T.Y., Lim H.Y., Kudo M., Breder V., Merle P. (2022). Updated efficacy and safety data from IMbrave150: Atezolizumab plus bevacizumab vs. sorafenib for unresectable hepatocellular carcinoma. J. Hepatol..

[82] Abou-Alfa G.K., Lau G., Kudo M., Chan S.L., Kelley R.K., Furuse J., Sukeepaisarnjaroen W., Kang Y.K., Van Dao T., De Toni E.N. (2022). Tremelimumab plus Durvalumab in Unresectable Hepatocellular Carcinoma. NEJM Evid..

[83] Sangro B., Chan S.L., Kelley R.K., Lau G., Kudo M., Sukeepaisarnjaroen W., Yarchoan M., De Toni E.N., Furuse J., Kang Y.K. (2024). Four-year overall survival update from the phase III HIMALAYA study of tremelimumab plus durvalumab in unresectable hepatocellular carcinoma. Ann. Oncol..

[84] Yau T., Galle P.R., Decaens T., Sangro B., Qin S., da Fonseca L.G., Karachiwala H., Blanc J.F., Park J.W., Gane E. (2025). Nivolumab plus ipilimumab versus lenvatinib or sorafenib as first-line treatment for unresectable hepatocellular carcinoma (CheckMate 9DW): An open-label, randomised, phase 3 trial. Lancet.

[85] Ilagan C.H., Goldman D.A., Gönen M., Aveson V.G., Babicky M., Balachandran V.P., Drebin J.A., Jarnagin W.R., Wei A.C., Kingham T.P. (2022). Recurrence of Hepatocellular Carcinoma After Complete Radiologic Response to Trans-arterial embolization: A Retrospective Study on Patterns, Treatments, and Prognosis. Ann. Surg. Oncol..

[86] Meyer T., Fox R., Ma Y.T., Ross P.J., James M.W., Sturgess R., Stubbs C., Stocken D.D., Wall L., Watkinson A. (2017). Sorafenib in combination with transarterial chemoembolisation in patients with unresectable hepatocellular carcinoma (TACE 2): A randomised placebo-controlled, double-blind, phase 3 trial. Lancet Gastroenterol. Hepatol..

[87] Kudo M., Imanaka K., Chida N., Nakachi K., Tak W.Y., Takayama T., Yoon J.H., Hori T., Kumada H., Hayashi N. (2011). Phase III study of sorafenib after transarterial chemoembolisation in Japanese and Korean patients with unresectable hepatocellular carcinoma. Eur. J. Cancer.

[88] Kudo M., Ren Z., Guo Y., Han G., Lin H., Zheng J., Ogasawara S., Kim J.H., Zhao H., Li C. (2025). Transarterial chemoembolisation combined with lenvatinib plus pembrolizumab versus dual placebo for unresectable, non-metastatic hepatocellular carcinoma (LEAP-012): A multicentre, randomised, double-blind, phase 3 study. Lancet.

[89] Sangro B., Kudo M., Erinjeri J.P., Qin S., Ren Z., Chan S.L., Arai Y., Heo J., Mai A., Escobar J. (2025). Durvalumab with or without bevacizumab with transarterial chemoembolisation in hepatocellular carcinoma (EMERALD-1): A multiregional, randomised, double-blind, placebo-controlled, phase 3 study. Lancet.

[90] Dong J., Han G., Ogasawara S., Liu R., Gu S., Liu F., Zhao M., Hu H., Liu Z., Lin K. (2025). LBA2 TALENTACE: A phase III, open-label, randomized study of on-demand transarterial chemoembolization (TACE) combined with atezolizumab + bevacizumab (Atezo+Bev) or on-demand TACE alone in patients with systemically untreated, intermediate-to-high burden unresectable hepatocellular carcinoma (uHCC). Ann. Oncol..

[91] Tanaka H., Kubo S., Tsukamoto T., Shuto T., Takemura S., Yamamoto T., Okuda T., Kanazawa A., Hirohashi K. (2005). Recurrence rate and transplantability after liver resection in patients with hepatocellular carcinoma who initially met transplantation criteria. Transpl. Proc..

[92] Bruix J., Takayama T., Mazzaferro V., Chau G.Y., Yang J., Kudo M., Cai J., Poon R.T., Han K.H., Tak W.Y. (2015). Adjuvant sorafenib for hepatocellular carcinoma after resection or ablation (STORM): A phase 3, randomised, double-blind, placebo-controlled trial. Lancet Oncol..

[93] Qin S., Chen M., Cheng A.L., Kaseb A.O., Kudo M., Lee H.C., Yopp A.C., Zhou J., Wang L., Wen X. (2023). Atezolizumab plus bevacizumab versus active surveillance in patients with resected or ablated high-risk hepatocellular carcinoma (IMbrave050): A randomised, open-label, multicentre, phase 3 trial. Lancet.

